# Haploinsufficiency of the Tyrosine Hydroxylase Gene in the Inbred C57BL/6J Strain Alters Behavior, Immunity, and Oxidative Stress, Especially After Acute Stress

**DOI:** 10.3390/ijms26188818

**Published:** 2025-09-10

**Authors:** Judith Félix, Antonio Garrido, Mónica De la Fuente

**Affiliations:** 1 Department of Genetics, Physiology and Microbiology (Animal Physiology Unit), School of Biological Sciences, Complutense University of Madrid, 28040 Madrid, Spain; mondelaf@ucm.es; 2 Research Institute of the Hospital 12 de Octubre, 28040 Madrid, Spain; 3 Nanocaging Research Group, Department of Biosciences, School of Biomedical and Health Sciences, Universidad Europea de Madrid, Villaviciosa de Odón, 28670 Madrid, Spain

**Keywords:** restraint stress, tyrosine hydroxylase, behavior, immunity, oxidative stress, C57BL/6J strain

## Abstract

Catecholamines (CA) are considered to play key roles in acute stress responses, but they also regulate important functions of the nervous, immune, and endocrine systems and are essential for body homeostasis and health. In Swiss mice (an outbred strain) with haploinsufficiency of the tyrosine hydroxylase gene (*Th*, TH-HZ), which encodes the rate-limiting enzyme of catecholamine synthesis, impairments in homeostatic system functions and a reduced lifespan have been reported. Moreover, these homeostatic alterations are exacerbated when these animals are exposed to acute restraint stress. Nonetheless, the effects of this genetic modification on an inbred strain, such as C57BL/6J, are undetermined. Given that the genetic background of mice can affect the phenotype of any genetic modification, this work aimed to characterize how behavioral responses, immunity, and the oxidative state in C57BL/6J mice are altered by *Th* haploinsufficiency under basal conditions after being subjected to 10 min of acute restraint stress. Sex differences were also considered. Compared with their WT counterparts, TH-HZ C57BL/6J animals exhibit behavioral impairments, immunosenescence, and oxidative stress under basal conditions. After stress, TH-HZ animals (both sexes) exhibit deteriorated behavior and immune functions. Therefore, *Th* haploinsufficiency in the inbred C57BL/6J strain triggers impairments in behavior, immunity, and the redox state. These findings corroborate the role of CA in maintaining regulatory system functions and highlight the importance of mouse strains in basic research.

## 1. Introduction

Catecholamines (CA) are widely known for their influence on the acute stress response through the neuroendocrine communication provided by the sympathetic–adrenomedullary axis (SAM) [1,2,3]. However, these neurohormones regulate other physiological processes that are essential for the adequate function of the organism’s regulatory systems, whose maintenance is vital for homeostasis and, hence, maintaining health [1].

CA critically modulate neural processes underlying cognition, attention, and memory, while also contributing to the regulation of emotional states. In particular, they buffer anxiety-related responses and facilitate appropriate locomotor control [4,5].

In the context of immunity, these mediators are important because they regulate innate and adaptive immune functions, exerting anti-inflammatory effects [6,7]. Based on these findings, the idea that presenting correct amounts of CA is vital for maintaining the homeostasis of the organism and health arises, and these adequate levels consequently reduce the morbidity and mortality of individuals [8,9,10].

Several years ago, an outbred Swiss mouse model exhibiting premature aging was developed and characterized, based on haploinsufficiency of the tyrosine hydroxylase (*Th*) gene, which serves as the rate-limiting enzyme in catecholamine synthesis. These mice, which contain only one allele for the gene that encodes *Th* (TH-HZ), present low production of CA, hindering the correct functioning of homeostatic systems.

In fact, their nervous function is impaired, and they exhibit increased anxiety-like behaviors and reduced exploratory and sensorimotor abilities. Additionally, they present a deterioration of several immune functions, such as those changes observed in chronologically aged mice [11,12], a process known as immunosenescence [11,13], and associated with a high degree of oxidation [13,14].

Taken together, these impairments lead to a loss of homeostasis, resulting in poorer health and a reduced lifespan [8,9,10]. These animals exhibit a significantly shorter lifespan than their wild-type counterparts [8].

In addition, when these animals are subjected to acute restraint stress, they cannot achieve an adequate response, resulting in severe impairments of their regulatory systems compared with their deterioration before this challenge. Moreover, their WT counterparts exhibit a good adaptive response to this stress [10].

The genetic background of laboratory rodents has important implications for scientific outcomes [15]. Accumulating evidence indicates that although high levels of genetic variation, as seen in outbred strains, can reduce experimental power and increase variability in treatment responses, the findings may better reflect human populations.

In contrast, inbred strains offer greater experimental power and require fewer animals by minimizing noise from segregating genetic variants [16,17]. Considering these genetic differences, the same experimental design employing different strains can yield opposite results, increasing the degree of inbreeding as a determining factor in the development of experimental procedures [18,19,20].

Classically, C57BL/6 mice have been preferred as an inbred strain for neurobehavioral analyses and immunological studies (to prevent alloimmune responses). However, because of their low genetic variability, these animals exhibit high hyperreactivity to new environments, altered behavioral responses, and elevated rates of exploration together with immune peculiarities, which can profoundly affect their nervous and immune responses to genetic modifications [21,22,23].

Notably, recent studies have shown that C57BL/6J mice exhibit sex-dependent variations in their behavioral and immunological responses to stress, highlighting the importance of considering both male and female animals when evaluating stress-related outcomes [24]. These differences can affect locomotor activity, anxiety-like behaviors, and immune function, and may interact with underlying genetic modifications. Understanding how *Th* haploinsufficiency influences these responses in a controlled inbred background is therefore essential for accurately interpreting the effects of catecholamine deficiency on homeostasis. By integrating strain- and sex-specific responses into the experimental design, the study aims to provide a more comprehensive characterization of stress-related behavioral and immune phenotypes.

Accordingly, notable phenotypic differences have been reported in comparisons of the consequences of several genetic alterations when inbred and outbred strains are employed to develop genetically engineered mice (GEMs) [25,26,27,28]. While Swiss GEMs are affected by genetic alterations, the same genetic modification of pure C57BL/6 GEMs resulted in minimal changes [29,30,31,32,33].

These controversial findings have also revealed how mice cope with stressful situations. Inbred mice, such as the BALB/c strain, seem more resistant to stressful situations, whereas outbred animals, such as Swiss mice, are affected by these environmental challenges [34].

Therefore, considering that the possible effects of *Th* haploinsufficiency on nervous and immune system functions in C57BL/6J mice are undetermined and considering the impact of strain-related variations previously reported under basal and stress conditions, the present work aimed to determine the effects of this genetic alteration on different behavioral, immune function, and redox state parameters in TH-HZ inbred C57BL/6J mice (male and female) under both conditions.

## 2. Results

### 2.1. Behavioral Trials

**Figure 1:**
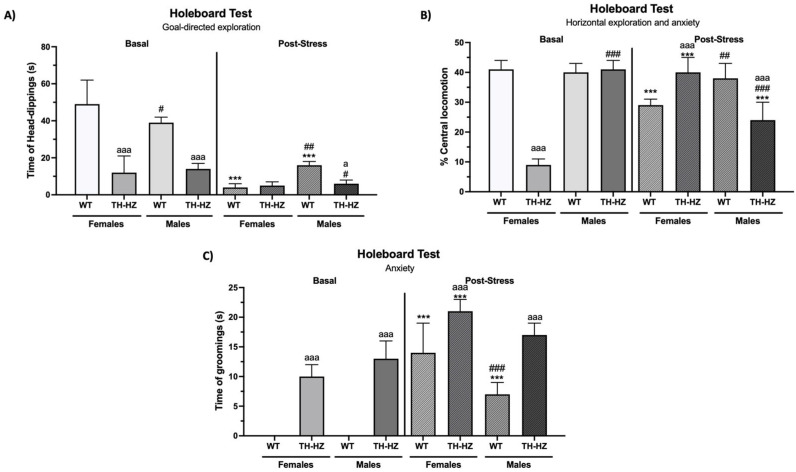
Exploratory and anxiety-like behaviors. (**A**) Time (in seconds) performing head-dippings in the holeboard test. (**B**) % Central locomotion in the holeboard test. (**C**) Time (in seconds) performing groomings in the holeboard test. Each column shows the mean ± standard deviation (*n* = 6). a *p* < 0.05, aaa *p* < 0.001 compared to WT. # *p* < 0.05, ## *p* < 0.01, ### *p* < 0.001 compared to female. *** *p* < 0.001 compared to basal condition.

We determined the differences between WT and TH-HZ female and male mice, by performing a battery of behavioral tests in each experimental group (females: WT and TH-HZ; males: WT and TH-HZ). The test battery comprised visual placing and hindlimb extensor reflex assessments, as well as the wood rod, tightrope, holeboard, T-maze, corner, and marble-burying tests. These tests were repeated after restraint stress to study the acute stress response of the mice. The results are shown in Figure 1, Table 1 and Table 2, and Supplementary Tables S2 and S3.

#### 2.1.1. Sensorimotor Abilities

Regarding the sensorimotor abilities, ANOVA revealed that genotype had a significant effect on freezing time (F(1,20) = 265.8, *p* < 0.001) but not on the number of freezing events (F(1,20) = 0, *p* > 0.999).

The same result was observed for the sex factor (F(1,20) = 265.8, *p* < 0.001; F(1,20) = 0, *p* > 0.999, respectively).

With respect to the repeated measures (stress), effects on freezing time (F(1,20) = 46.15, *p* < 0.001) and the number of freezing cycles (F(1,20) = 43.2, *p* < 0.001) were observed.

A significant interaction effect between genotype and sex was also observed for the freezing time (F(1,20) = 265.8, *p* < 0.001) and number of freezing events (F(1,20) = 172.8, *p* < 0.001), as well as interaction effects between genotype and stress (F(1,20) = 46.15, *p* < 0.001; F(1,20) = 43.2, *p* < 0.001, respectively) and between sex and stress (F(1,20) = 46.15, *p* < 0.001; F(1,20) = 43.2 *p* < 0.001, respectively).

Finally, when the interaction between genotype, sex, and stress was studied, it was significant for both freezing time (F(1,20) = 46.15, *p* < 0.001) and number of freezing behaviors (F(1,20) = 43.2, *p* < 0.001).

##### Post Hoc Analysis

*Basal condition:* The post hoc analysis showed that, under basal conditions, TH-HZ females exhibited more freezing behaviors and spent more time performing the wood rod test (Table 1, *p* < 0.001) than WT females. TH-HZ males exhibited fewer freezing behaviors and spent less time performing the wood rod test (Table 1, *p* < 0.001) than did TH-HZ females.

*Post-stress condition:* When we analyzed genotype and sex differences under post-stress conditions, we observed that TH-HZ females performed more freezing behaviors and spent more time performing the wood rod test than WT females did (Table 1, *p* < 0.001). In terms of sex differences under post-stress conditions, TH-HZ males froze less frequently and spent less time performing the wood rod test (Table 1, *p* < 0.001) than did TH-HZ females.

*Effects of stress:* Finally, an analysis of the effects of stress revealed that TH-HZ females spent more time performing the wood rod test (Table 1, *p* < 0.001) than under the basal condition.

#### 2.1.2. Exploratory and Anxiety-like Behaviors

##### Holeboard Test

When exploration and anxiety behaviors were assessed using the holeboard test, ANOVA revealed that the genotype had a significant effect on the number and timing of central rearing behaviors (F(1,20) = 76.8, *p* < 0.001; F(1,20) = 120.3, *p* < 0.001, respectively), the percentages of central (F(1,20) = 57.32, *p* < 0.001) and peripheral locomotion (F(1,20) = 34.42, *p* < 0.001), the number and duration of grooming (F(1,20) = 132.5, *p* < 0.001; F(1,20) = 192, *p* < 0.001, respectively), the number and duration of freezing (F(1,20) = 61.89, *p* < 0.001; F(1,20) = 88.62, *p* < 0.001, respectively), and the number and duration of head dips (F(1,20) = 63.13, *p* < 0.001; F(1,20) = 180.9, *p* < 0.001, respectively).

With respect to the sex factor, no significant effect was observed on the number and time of central rearing behaviors (F(1,20) = 0, *p* > 0.999; F(1,20) = 0.33, *p* = 0.566, respectively) or on the percentage of peripheral locomotion (F(1,20) = 3.82, *p* = 0.057), but significant effects on the percentage of central locomotion (F(1,20) = 28.56, *p* < 0.001), on the number and time of grooming (F(1,20) = 14.73, *p* < 0.001; F(1,20) = 7.68, *p* < 0.001, respectively), on the number and time of freezing (F(1,20) = 20.21, *p* < 0.001; F(1,20) = 9.85, *p* = 0.003, respectively), and on the number and time of head dips (F(1,20) = 4.69, *p* = 0.036; F(1,20) = 4.89, *p* = 0.032, respectively) were observed.

The repeated measure (stress) had an effect on the number and time of central rearing behaviors (F(1,20) = 76.8, *p* < 0.001; F(1,20) = 120.3, *p* < 0.001, respectively), the number and time of grooming (F(1,20) = 132.5, *p* < 0.001; F(1,20) = 155.5, *p* < 0.001, respectively), the number and time of freezing (F(1,20) = 102.3, *p* < 0.001; F(1,20) = 138.5, *p* < 0.001, respectively), and the number and time of head dips (F(1,20) = 25.57, *p* < 0.001; F(1,20) = 163, *p* < 0.001, respectively); however, it had no effect on the percentage of central or peripheral locomotion (F(1,20) = 0, *p* > 0.999; F(1,20) = 2.06, *p* = 0.159, respectively). When the effect of interactions was studied, the interaction between genotype and sex was significant for the percentage of peripheral locomotion (F(1,20) = 6.14, *p* = 0.017), grooming time (F(1,20) = 4.32, *p* = 0.044), number and time of freezing (F(1,20) = 11.37, *p* = 0.002; F(1,20) = 9.85, *p* = 0.003, respectively), and number of head dips (F(1,20) = 42.26, *p* < 0.001).

The interaction effect between genotype and stress was significant for the number and time of central rearing behaviors (F(1,20) = 76.8, *p* < 0.001; F(1,20) = 120.3, *p* < 0.001, respectively), for the percentages of central (F(1,20) = 38.88, *p* < 0.001) and peripheral locomotion (F(1,20) = 6.14, *p* = 0.017), for the number and time of grooming (F(1,20) = 4.55, *p* = 0.04; F(1,20) = 4.32, *p* = 0.04, respectively), for the number and time of freezing (F(1,20) = 20.21, *p* < 0.001; F(1,20) = 15.38, *p* < 0.001, respectively), and for the number and time of head dips (F(1,20) = 13.04, *p* = 0.001; F(1,20) = 107.9, *p* < 0.001, respectively).

The interaction effect between sex and stress was significant for the percentages of central and peripheral locomotion (F(1,20) = 71.6, *p* < 0.001; F(1,20) = 40.81, *p* < 0.001, respectively), for the number and duration of grooming (F(1,20) = 8.91, *p* = 0.004; F(1,20) = 23.52, *p* < 0.001, respectively), for freezing time (F(1,20) = 5.54, *p* = 0.023), and for the number and duration of head dipping (F(1,20) = 42.26, *p* < 0.001; F(1,20) = 4.89, *p* = 0.032).

Finally, when the interaction between genotype, sex, and stress was studied, it was significant for the percentages of central (F(1,20) = 166.8, *p* < 0.001) and peripheral locomotion (F(1,20) = 40.81, *p* < 0.001), for freezing time (F(1,20) = 5.54, *p* = 0.023), and for the number and time of head dips (F(1,20) = 13.04, *p* = 0.001; F(1,20) = 27.86, *p* < 0.001, respectively).

##### T-Maze

With respect to the T-maze, ANOVA revealed that genotype had an effect on exploratory efficacy (F(1,20) = 31.17, *p* < 0.001), the number and duration of grooming behaviors (F(1,20) = 54, *p* < 0.001; F(1,20) = 147, *p* < 0.001, respectively), and the number and duration of freezing (F(1,20) = 150, *p* < 0.001; F(1,20) = 150, *p* < 0.001, respectively).

The sex factor affected the number and duration of grooming behaviors (F(1,20) = 6, *p* = 0.018; F(1,20) = 75, *p* < 0.001, respectively), and the number and duration of freezing (F(1,20) = 150, *p* < 0.001; F(1,20) = 150, *p* < 0.001, respectively).

The dependent variable (stress) had effects on exploratory efficacy (F(1,20) = 3.92, *p* = 0.049), the number and time of grooming behaviors (F(1,20) = 96, *p* < 0.001; F(1,20) = 56.33, *p* < 0.001, respectively), and the number and time of freezing (F(1,20) = 150, *p* < 0.001; F(1,20) = 150, *p* < 0.001, respectively).

The interaction effect between sex and genotype was significant for the number and duration of grooming (F(1,20) = 6, *p* = 0.018; F(1,20) = 56.33, *p* < 0.001, respectively), and for the number and duration of freezing (F(1,20) = 150, *p* < 0.001; F(1,20) = 150, *p* < 0.001, respectively).

The interaction effect between genotype and stress was significant for exploratory efficacy (F(1,20) = 19.2, *p* < 0.001), the number and timing of grooming behaviors (F(1,20) = 24, *p* < 0.001; F(1,20) = 16.33, *p* < 0.001, respectively), and the number and timing of freezing (F(1,20) = 150, *p* < 0.001; F(1,20) = 150, *p* < 0.001, respectively).

The interaction effect between sex and stress was significant only for the number of freezing behaviors and the duration of freezing (F(1,20) = 150, *p* < 0.001; F(1,20) = 150, *p* < 0.001, respectively).

Finally, when the interaction between genotype, sex, and stress was studied, it was significant for the number and duration of freezing (F(1,20) = 150, *p* < 0.001; F(1,20) = 150, *p* < 0.001).

##### Marble-Burying Test

Finally, with respect to the marble-burying test, ANOVA revealed that genotype had effects on the numbers of intact, moved, and buried pieces under the standard condition (F(1,20) = 145.2, *p* < 0.001; F(1,20) = 21, *p* < 0.001; F(1,20) = 66, *p* < 0.001, respectively), and on the numbers of intact, moved, and buried pieces under the bizonal condition (F(1,20) = 106.9, *p* < 0.001; F(1,20) = 50.82, *p* < 0.001; F(1,20) = 46.15, *p* < 0.001, respectively).

The sex factor had effects on the number of intact pieces under the standard condition (F(1,20) = 4.8, *p* = 0.034), and on the numbers of intact, moved, and buried pieces under the bizonal condition (F(1,20) = 19.64, *p* < 0.001; F(1,20) = 5.65, *p* = 0.022; F(1,20) = 7.39, *p* = 0.009, respectively).

The dependent variable (stress) had effects on the numbers of pieces moved and buried under the standard condition (F(1,20) = 21, *p* < 0.001; F(1,20) = 4.91, *p* = 0.032, respectively), and on the numbers of intact, moved, and buried pieces under the bizonal condition (F(1,20) = 8.73, *p* = 0.005; F(1,20) = 5.65, *p* = 0.022; F(1,20) = 7.39, *p* = 0.009, respectively).

The interaction effect between sex and genotype was significant for the numbers of intact, moved, and buried pieces under the standard condition (F(1,20) = 4.8, *p* = 0.034; F(1,20) = 51.86, *p* < 0.001; F(1,20) = 4.91, *p* = 0.032, respectively), and for the numbers of intact and buried pieces under the bizonal condition (F(1,20) = 8.73, *p* = 0.005; F(1,20) = 7.39, *p* = 0.009, respectively).

The interaction effect between sex and stress was significant for the number of pieces moved under the standard condition (F(1,20) = 10.71, *p* = 0.002).

The interaction effect between genotype and stress was not significant for any of the parameters, nor was the interaction effect between genotype, sex, and stress (Supplementary Table S4).

##### Post Hoc Analysis

*Basal condition:* The post hoc analysis of the results of the holeboard test under the basal condition revealed that TH-HZ females exhibited less central locomotion (Figure 1B, *p* < 0.001), more peripheral locomotion (Table 2, *p* < 0.001), and head dipping for less time (Figure 1A, *p* < 0.001); spent more time performing grooming (Figure 1C, *p* < 0.001); performed fewer rearing behaviors (Table 2, *p* < 0.001); and spent more time freezing (Table 2, *p* < 0.001) than did WT females. In addition, under the basal condition, TH-HZ females spent more time grooming in the T-maze test (Table 2, *p* < 0.001), moved more pieces under the standard and bizonal conditions of the marble-burying test (Table 2, *p* < 0.001), and buried more pieces under the bizonal condition (Table 2, *p* < 0.001) than did WT females.

Under basal conditions, compared with WT males, TH-HZ males spent more time performing grooming (Figure 1C, *p* < 0.001), less time performing head dips (Figure 1A, *p* < 0.001), and did not rear (Table 2, *p* < 0.001) in the holeboard test. In addition, compared with WT males, TH-HZ males left fewer pieces intact (Table 2, *p* < 0.001) and buried more pieces (Table 2, *p* = 0.002) under the standard conditions of the marble-burying test and moved more pieces under bizonal conditions (Table 2, *p* = 0.019).

In terms of sex, no differences were detected in the WT mice under the basal condition. However, TH-HZ males exhibited more central locomotion (Figure 1B, *p* < 0.001) and less peripheral locomotion (Table 2, *p* < 0.001), as well as less freezing time (Table 2, *p* < 0.001) in the holeboard test, and they moved more pieces under the standard condition of the marble-burying test (Table 2, *p* = 0.007) than did TH-HZ females in the basal condition.

*Post-stress condition:* Under post-stress conditions, TH-HZ females presented greater central locomotion (Figure 1B, *p* < 0.001) and spent more time performing grooming (Table 2, *p* < 0.001) and freezing behaviors (Table 2, *p* < 0.001) in the holeboard test than did WT females. In the T-maze, TH-HZ females took longer to complete the test (Table 2, *p* < 0.001) and performed more grooming and freezing behaviors (Table 2, *p* < 0.001) than WT females did. In the marble-burying test performed after exposure to stress, TH-HZ females moved more pieces under the standard condition (Table 2, *p* < 0.001), and buried more pieces under the bizonal condition (Table 2, *p* = 0.004) than WT females did.

However, in the post-stress condition, TH-HZ males presented less central locomotion (Figure 1B, *p* < 0.001), longer grooming and freezing times (Figure 1C, *p* < 0.001; Table 2, *p* < 0.001, respectively), and shorter head dips (Figure 1A, *p* = 0.023) than WT males did. Similarly, in the T-maze, post-stress TH-HZ males took longer to complete the test (Table 2, *p* < 0.001) and spent more time performing grooming (Table 2, *p* < 0.001) than WT males did. In the marble-burying test performed after exposure to stress, TH-HZ males presented fewer intact pieces and more buried pieces (Table 2, *p* < 0.001) than WT males did.

With respect to sex differences in the post-stress condition, WT males presented greater central locomotion (Figure 1B, *p* = 0.006), spent less time performing grooming behaviors (Figure 1C, *p* < 0.001), spent more time performing head dipping (Figure 1A, *p* < 0.001) in the holeboard test, and kept fewer pieces intact under the bizonal condition of the marble-burying test (Table 2, *p* = 0.002) than did WT females. For TH-HZ, post-stress males presented less central locomotion (Figure 1B, *p* < 0.001), more freezing (Table 2, *p* = 0.002) and longer head dips (Figure 1A, *p* < 0.049) in the holeboard test, as well as longer grooming and freezing times in the T-maze (Table 2, *p* < 0.001), and fewer pieces moved under the standard marble-burying test (Table 2, *p* < 0.001) than did TH-HZ females under the post-stress condition.

*Effects of stress:* Finally, with respect to the effects of stress on exploration and anxiety-like behaviors, WT females presented less central locomotion (Figure 1B, *p* < 0.001), longer grooming and freezing times (Figure 1C, *p* < 0.001; Table 2, *p* = 0.007, respectively), and shorter head dipping and rearing times (Figure 1A, *p* < 0.001; Table 2, *p* < 0.001, respectively) in the holeboard test than in the basal condition. After stress, WT males spent more time grooming and freezing (Figure 1C, *p* < 0.001; Table 2, *p* = 0.007, respectively), as well as presented fewer head dips and rearing behaviors (Figure 1A, *p* < 0.001; Table 2, *p* < 0.001, respectively), than under the basal condition.

The TH-HZ females presented greater central locomotion (Figure 1B, *p* < 0.001) and spent more time performing grooming and freezing in the holeboard test (Figure 1C, *p* < 0.001; Table 2, *p* < 0.001), and presented more grooming and freezing behaviors in the T-maze (Table 2, *p* < 0.001) than under the basal condition. Finally, TH-HZ males exhibited less central locomotion (Figure 1B, *p* < 0.001) and more grooming and freezing behaviors (Table 2, *p* < 0.001) in the holeboard test. They took longer to complete the test (Table 2, *p* < 0.001) and performed more grooming (Table 2, *p* < 0.001) in the T-maze test than under the basal condition.

### 2.2. Immune Function

**Figure 2:**
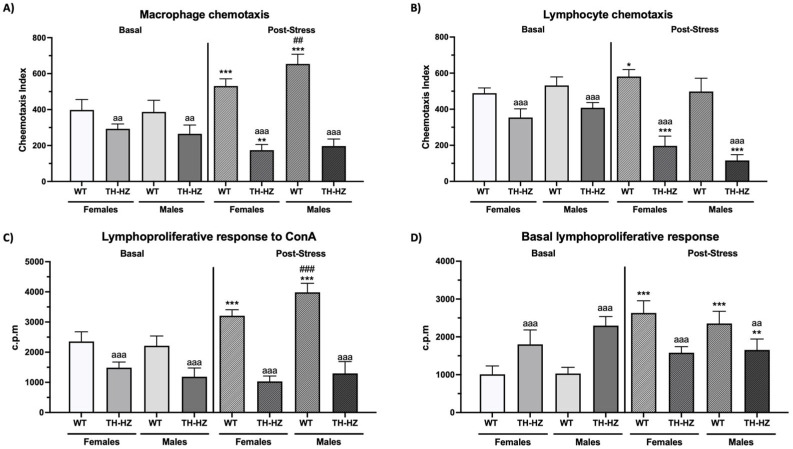
Immunity parameters. (**A**) Macrophage chemotaxis. (**B**) Lymphocyte chemotaxis. (**C**) Lymphoproliferative response to ConA (c.p.m). (**D**) Basal lymphoproliferative response (c.p.m). Each column shows the mean ± standard deviation (*n* = 6). aa *p* < 0.01, aaa *p* < 0.001 compared to WT. ## *p* < 0.01, ### *p* < 0.001 compared to female. * *p* < 0.05, ** *p* < 0.01, *** *p* < 0.001 compared to basal condition.

Data related to immune function are presented in Figure 2 and Table 3.

When immune function parameters were evaluated, ANOVA revealed that genotype had significant effects on macrophage chemotaxis (F(1,20) = 365.7, *p* < 0.001), lymphocyte chemotaxis (F(1,20) = 368, *p* < 0.001), natural killer activity (F(1,20) = 14.64, *p* < 0.001), lymphoproliferation in response to lipopolysaccharide (F(1,20) = 678.5, *p* < 0.001), and concanavalin A (F(1,20) = 426.7, *p* < 0.001).

Sex had effects on natural killer activity (F(1,20) = 146.8, *p* < 0.001) and on the lymphoproliferative response to lipopolysaccharide (F(1,20) = 1.21, *p* = 0.028).

The dependent variable (stress) had effects on macrophage and lymphocyte chemotaxis (F(1,20) = 15.31, *p* < 0.001; F(1,20) = 53.54, *p* < 0.001, respectively), natural killer activity (F(1,20) = 19.93; *p* < 0.001), basal lymphoproliferation (F(1,20) = 43.57, *p* < 0.001), and the response to lipopolysaccharide and concanavalin A (F(1,20) = 14.46, *p* < 0.001; F(1,20) = 48.65, *p* < 0.001, respectively).

The interaction effect between genotype and sex was significant for macrophage chemotaxis (F(1,20) = 4.62, *p* = 0.037), natural killer activity (F(1,20) = 14.64, *p* < 0.001), basal lymphoproliferation (F(1,20) = 6.87, *p* = 0.012), and lymphoproliferation in response to concanavalin A (F(1,20) = 4.22, *p* = 0.049). The interaction effect between genotype and stress was significant for macrophage and lymphocyte chemotaxis (F(1,20) = 116.3, *p* < 0.001; F(1,20) = 90.02, *p* < 0.001, respectively), natural killer activity (F(1,20) = 104.1, *p* < 0.001), and lymphoproliferation under basal and LPS- and ConA-stimulated conditions (F(1,20) = 146.3, *p* < 0.001; F(1,20) = 46.93, *p* < 0.001; F(1,20) = 82.19, *p* < 0.001, respectively).

The interaction effect between sex and stress was significant for macrophage and lymphocyte chemotaxis (F(1,20) = 11.55, *p* = 0.002; F(1,20) = 23.86, *p* < 0.001, respectively), natural killer activity (F(1,20) = 49.22, *p* < 0.001), and lymphoproliferation under basal and ConA-stimulated conditions (F(1,20) = 5.25, *p* = 0.027; F(1,20) = 20.58, *p* < 0.001, respectively). The interaction effect between genotype, sex, and stress was significant for natural killer activity (F(1,20) = 6.51, *p* = 0.014).

#### Post Hoc Analysis

*Basal condition:* Several differences were observed in the post hoc analysis. Under basal conditions, TH-HZ females and males exhibited lower chemotaxis of macrophages (Figure 2A, *p* = 0.008; *p* = 0.002, respectively) and lymphocytes (Figure 2B, *p* < 0.001), as well as a lower lymphoproliferative response to concanavalin A (Figure 2C, *p* < 0.001) and polysaccharide (Table 3, *p* < 0.001) mitogens and greater basal lymphoproliferation (Figure 2D, *p* < 0.001) than did WT mice. Compared with WT males, TH-HZ males also presented greater natural killer activity (Table 3, *p* = 0.009). Under basal conditions, no sex differences were detected in either the WT or TH-HZ mice.

*Post-stress condition:* Under post-stress conditions, TH-HZ females and males presented lower macrophage (Figure 2A, *p* < 0.001) and lymphocyte (Figure 2B, *p* < 0.001) chemotaxis, lower natural killer activity (Table 3, *p* < 0.001; *p* = 0.009, respectively), and a lower proliferative response under basal conditions (Figure 2D, *p* < 0.001; *p* = 0.002, respectively), and following concanavalin A (Figure 2C, *p* < 0.001) and lipopolysaccharide stimulation (Table 3, *p* < 0.001) than WT mice. With respect to sex differences, WT males presented greater macrophage chemotaxis (Figure 2A, *p* = 0.002), lower natural killer activity (Table 3, *p* < 0.001), and a greater proliferative response to concanavalin A (Figure 2C, *p* < 0.001) than WT females did. The TH-HZ males presented lower natural killer activity (Table 3, *p* < 0.001) than did the TH-HZ females under post-stress conditions.

*Effects of stress:* Finally, when the effects of stress were evaluated, a general improvement in immunity was observed in WT mice, and a deterioration was observed in TH-HZ mice. WT females showed increases in macrophage (Figure 2A, *p* < 0.001) and lymphocyte (Figure 2B, *p* = 0.026) chemotaxis, improved NK activity (Table 3, *p* < 0.001), increased basal lymphoproliferation (Figure 2D, *p* < 0.001), and lymphoproliferation in response to concanavalin A (Figure 2C, *p* < 0.001) and lipopolysaccharide (Table 3, *p* = 0.015) compared to the basal condition. In WT males, increased macrophage chemotaxis (Figure 2A, *p* < 0.001) and lymphoproliferation were observed under basal (Figure 2D, *p* < 0.001), ConA (Figure 2C, *p* < 0.001), and LPS (Table 3, *p* < 0.001) stimulation conditions compared with the basal conditions.

When we observed TH-HZ females after stress, they exhibited impaired macrophage (Figure 2A, *p* < 0.001) and lymphocyte (Figure 2B, *p* < 0.001) chemotaxis as well as NK activity (Table 3, *p* < 0.001) compared with those under basal conditions. Furthermore, TH-HZ males presented impaired lymphocyte chemotaxis (Figure 2B, *p* < 0.001) and NK activity (Table 3, *p* < 0.001), as well as decreased basal proliferation (Figure 2D, *p* = 0.005), compared with the basal conditions.

### 2.3. Oxidative Stress

**Figure 3:**
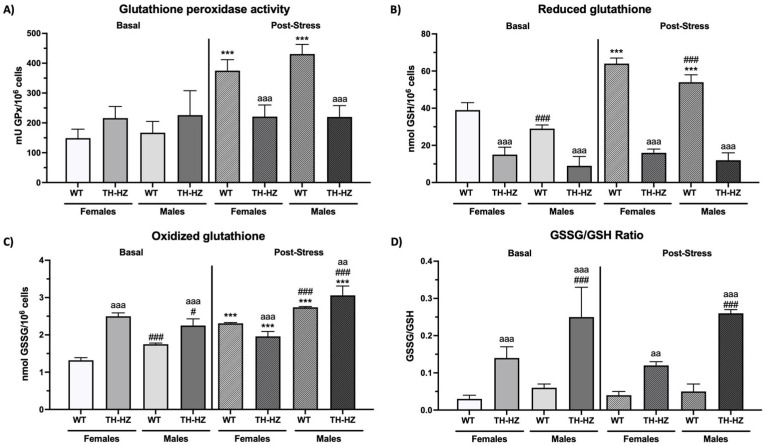
Oxidative stress parameters. (**A**) Glutathione peroxidase activity (mU GPx/10^6^ cells). (**B**) Reduced glutathione levels (nmol GSH/10^6^ cells). (**C**) Oxidized glutathione levels (nmol GSSG/10^6^ cells). (**D**) GSSG/GSH Ratio. Each column shows the mean ± standard deviation (*n* = 6). aa *p* < 0.01, aaa *p* < 0.001 compared to WT. # *p* < 0.05, ### *p* < 0.001 compared to female. *** *p* < 0.001 compared to basal condition.

The data related to oxidative stress parameters are shown in Figure 3 and Table 4.

Regarding the oxidative stress parameters, ANOVA revealed that genotype had significant effects on catalase activity (F(1,20) = 67.85, *p* < 0.001), glutathione reductase activity (F(1,20) = 28.59, *p* < 0.001), glutathione peroxidase activity (F(1,20) = 21.49, *p* < 0.001), reduced glutathione levels (GSH) (F(1,20) = 1016, *p* < 0.001), xanthine oxidase activity (F(1,20) = 43.58, *p* < 0.001), oxidized glutathione levels (GSSG) (F(1,20) = 129.1, *p* < 0.001), and the GSSG/GSH ratio (F(1,20) = 254.7, *p* < 0.001).

The effect of sex was significant for glutathione reductase activity (F(1,20) = 9.41, *p* = 0.004), GSH levels (F(1,20) = 50.94, *p* < 0.001), GSSG levels (F(1,20) = 138.7, *p* < 0.001), and the GSSG/GSH ratio (F(1,20) = 61.54, *p* < 0.001).

The dependent variable (stress) had effects on catalase activity (F(1,20) = 12.46, *p* = 0.001), glutathione peroxidase activity (F(1,20) = 89.97, *p* < 0.001), GSH levels (F(1,20) = 165.1, *p* < 0.001), xanthine oxidase activity (F(1,20) = 88.1, *p* < 0.001), and GSSG levels (F(1,20) = 240.1, *p* < 0.001).

The interaction between genotype and sex was significant for catalase (F(1,20) = 26, *p* < 0.001) and glutathione reductase (F(1,20) = 7.38, *p* = 0.009) activities, for GSH levels (F(1,20) = 5.66, *p* = 0.022), for xanthine oxidase activity (F(1,20) = 25.66, *p* < 0.001), and for the GSSG/GSH ratio (F(1,20) = 32.27, *p* < 0.001).

The interaction between genotype and stress had significant effects on catalase activity (F(1,20) = 18.62, *p* < 0.001), glutathione reductase and peroxidase activities (F(1,20) = 12.91, *p* = 0.001; F(1,20) = 90.71, *p* < 0.001, respectively), GSH levels (F(1,20) = 119.8, *p* < 0.001), xanthine oxidase activity (F(1,20) = 116.2, *p* < 0.001), and GSSG levels (F(1,20) = 138.7, *p* < 0.001).

The interaction effect between sex and stress was significant for xanthine oxidase activity (F(1,20) = 29.17, *p* < 0.001) and GSSG levels (F(1,20) = 86.44, *p* < 0.001). Finally, the interaction between genotype, sex, and stress had significant effects on catalase (F(1,20) = 18.62, *p* < 0.001) and glutathione reductase (F(1,20) = 5.6, *p* = 0.023) activities, xanthine oxidase activity (F(1,20) = 45.5, *p* < 0.001), and GSSG levels (F(1,20) = 86.44, *p* < 0.001).

#### Post Hoc Analysis

*Basal condition:* The post hoc analysis revealed that under basal conditions, female and male TH-HZ mice presented lower reduced glutathione concentrations (Figure 3B, *p* < 0.001), higher oxidized glutathione levels (Figure 3C, *p* < 0.001), and a lower GSSG/GSH ratio (Figure 3D, *p* < 0.001) than WT mice. Regarding sex differences, under basal conditions, WT males presented lower GSH levels (Figure 3B, *p* < 0.001) and higher GSSG (Figure 3C, *p* < 0.001) concentrations than WT females. However, TH-HZ males presented lower GSSG concentrations (Figure 3C, *p* = 0.027) and a higher GSSG/GSH ratio (Figure 3D, *p* < 0.001) than did TH-HZ females.

*Post-stress condition:* Under post-stress conditions, TH-HZ females presented lower catalase activity (Table 4, *p* < 0.001), lower glutathione peroxidase activity (Figure 3A, *p* < 0.001), lower GSH (Figure 3B, *p* < 0.001) and GSSG (Figure 3C, *p* < 0.001) concentrations, and a lower GSSG/GSH ratio (Figure 3D, *p* = 0.002) than WT females. Compared with WT males, TH-HZ males presented lower glutathione reductase activity (Table 4, *p* < 0.001) and GPx activity (Figure 3A, *p* < 0.001), lower GSH concentrations (Figure 3B, *p* < 0.001), lower xanthine oxidase activity (Table 4, *p* < 0.001), higher GSSG concentrations (Figure 3C, *p* = 0.002), and higher GSSG/GSH ratios (Figure 3D, *p* < 0.001). Furthermore, under post-stress conditions, WT males presented lower catalase activity (Table 4, *p* < 0.001), higher glutathione reductase activity (Table 4, *p* = 0.002), lower GSH levels (Figure 3B, *p* < 0.001), and higher GSSG concentrations (Figure 3C, *p* < 0.001) than WT females. Furthermore, TH-HZ males presented greater catalase activity (Table 4, *p* < 0.001), greater xanthine oxidase activity (Table 4, *p* < 0.001), higher GSSG concentrations (Figure 3C, *p* < 0.001), and higher GSSG/GSH ratios (Figure 3D, *p* < 0.001) than did WT females.

*Effects of stress:* Finally, when the effects of stress were studied, WT females presented increased catalase activity (Table 4, *p* < 0.001) and GPx activity (Figure 3A, *p* < 0.001), increased GSH concentrations (Figure 3B, *p* < 0.001), increased xanthine oxidase activity (Table 4, *p* < 0.001), and increased GSSG concentrations (Figure 3C, *p* < 0.001) with respect to the basal conditions. Compared with those under basal conditions, WT males subjected to stress presented increased GR activity (Table 4, *p* = 0.005) and GPx activity (Figure 3A, *p* < 0.001), increased GSH concentrations (Figure 3B, *p* < 0.001), increased XO activity (Table 4, *p* < 0.001), and increased GSSG concentrations (Figure 3D, *p* < 0.001). Compared with the basal condition, the XO activity of TH-HZ females and males increased (Table 4, *p* < 0.001), and the GSSG concentration decreased (Figure 3C, *p* < 0.001).

## 3. Discussion

This study is the first to characterize the behavioral response, immune function, and oxidative state of inbred C57BL/6J mice with haploinsufficiency of the tyrosine hydroxylase (*Th*) gene. We also analyzed the animals’ responses to restraint stress, highlighting the importance of CA in this response in this genetic background.

We began by comparing wild-type (WT) mice with mice carrying a single functional copy of the *Th* gene (TH-HZ) on the C57BL/6J background. For this purpose, we analyzed behavior, immune function, and the redox state under basal conditions (without stress).

In terms of behavior and, specifically, sensorimotor capacity, both male and female C57BL/6J TH-HZ mice presented less muscular vigor. Similarly, the TH-HZ mice of both sexes presented a lower exploratory capacity and greater anxiety-like behaviors. These results indicate the importance of CA in a wide variety of neurological functions, such as locomotor control and the anxiety response, as previously reported [4,35]. Other studies using animals with CA depletion due to *Th* deficiency reported similar results, with lower motor coordination and impaired sensorimotor abilities [36,37].

In the analysis of the immune system, TH-HZ animals exhibited impaired innate and adaptive immune responses, as indicated by decreased chemotaxis and proliferative capacity in the presence of mitogens. Moreover, they also showed greater basal proliferation, which indicates the establishment of a proinflammatory profile. Notably, CA modulate both acute and chronic inflammatory responses by promoting a shift from proinflammatory to anti-inflammatory cytokine profiles [38], thereby exerting anti-inflammatory effects [39,40]. Interestingly, other studies have shown that stress-induced immune alterations can be modulated by external factors, illustrating the broader context of stress–immunity interactions [41].

Although we did not analyze the plasma CA levels in C57BL/6J TH-HZ mice, this genetic alteration in Swiss mice triggers low levels of these neurotransmitters [9,42]. Considering these previous findings and the parallelism in some of the behavioral and immune alterations observed in the present work compared with previous findings, we hypothesized that C57BL/6J mice could have lower CA levels, like the animals employed with the same genetic alteration in previous studies (Swiss strain). Therefore, this inflammatory profile might reflect an inability to modulate the shift from a proinflammatory to an anti-inflammatory state. Nonetheless, future research should consider analyzing the CA levels of these C57BL/6J animals to corroborate this hypothetical mechanism.

Indeed, in TH-HZ mice of both sexes, decreases in the levels of antioxidant compounds accompanied by increases in oxidant levels were observed, which translated into the establishment of oxidative stress in these animals. This result could be considered controversial since previous reports described the oxidant capacity of CA for autooxidation, generating an increase in reactive free radicals [43,44,45].

However, this oxidant capacity is affected in a concentration-dependent manner, acting as a pro-oxidant at high concentrations [46,47], whereas at physiological values, CA exert antioxidant effects [47,48]. Taken together, these findings indicate that this genetic alteration in C57BL/6J mice also promotes accelerated aging-related consequences, as previously reported in outbred mice [8,9,10,42]. However, the impairments are less evident in the inbred strain employed in the present work. Importantly, previous reports have indicated that some of the behavioral, immune, and redox parameters analyzed in this work are good aging markers [12,49]. Although alterations in immune function and oxidative stress may be linked to changes in catecholamine signaling, it is also possible that other neurotransmitter systems contribute to these effects, highlighting the complexity of the consequences of *Th* haploinsufficiency.

Certain differences can be observed between inbred and outbred strains with this genetic alteration when the results obtained in this study are compared with those previously described in Swiss mice with the same genetic alteration [10]. In this sense, a less generalized deterioration of sensorimotor abilities was observed in C57BL/6J mice (as described above) than in Swiss mice (summarized in Supplementary Table S1).

The discrepancy in the results from the comparison of the consequences of the same genetic manipulation when inbred and outbred mouse strains are used to develop genetic engineering mice (GEMs) have also been reported by other authors. Tabuchi and colleagues reported that C57BL6/129S2/SvPasCrl hybrid mice harboring a mutation in the *Nlgn3* gene (NL3R451C) presented behavioral alterations, such as reduced social interactions [32]. Nonetheless, this same genetic modification in mice on a pure C57BL/6J background resulted in minimal alterations in behavior [33]. Similarly, the development of 5-HT1A receptor knockout mice on three different background strains (129Sv, C57BL/6, and Swiss mice) resulted in decreased sensitivity to benzodiazepines only in Swiss mice [29,30,31].

Although we do not know the reason for these strain discrepancies, one possibility could be the differential regulation of *Th* gene expression by the strain background [50], which seems to be greater in C57BL/6J mice than in the Swiss strain [51]. Nevertheless, future research to decipher the differences in the molecular regulation of the *Th* gene in these two strains should be performed to clarify this important aspect.

With respect to the impairments observed in immunity and the redox state, the effects of TH-HZ on Swiss and C57BL/6J mice are similar [8,9,10,42]. The major difference between these strains was observed when sex effects were studied. In this sense, C57BL/6J mice exhibited clear sexual dimorphism for some behaviors, such as several anxiety-like behavioral parameters (freezing and grooming) and locomotion.

With respect to immune function, this sexual effect was associated with natural killer activity and the lymphoproliferative response to ConA and LPS. Similarly, glutathione reductase activity, GSH and GSSG levels, and the ratios of both analytes also differed between male and female C57BL/6J mice (summarized in Supplementary Table S4).

However, in a study of the Swiss strain, great evident sexual dimorphism in TH-HZ animals was reported [10]. This differential trend depending on the mouse strain has also been reported in other genetically engineered mice (GEMs) [48]. Although the molecular mechanisms underlying these discrepancies are still unknown, one possible explanation could be the different responses to CA exhibited by males and females (being more significant in the former) [47], an effect that could be a consequence of differences in sympathetic nervous system regulation [49]. Taken together, these strain-related discrepancies in the effects of genetic manipulation highlight the importance of the strain chosen to develop GEMs.

Given the differences observed between strains under basal conditions, one would expect differences in their stress response. Therefore, we studied the effects of restraint stress for 10 min on WT and TH-HZ C57BL/6J mice.

The sensorimotor abilities of WT mice exposed to stress were unaffected. In contrast, an increase in anxiety and a decrease in exploratory ability were observed compared with those in the basal condition.

With respect to immune function, WT mice exhibited improved innate and adaptive responses. In the case of oxidative and inflammatory stress, WT mice presented increases in basal lymphoproliferation, the levels of oxidative compounds, and the levels of antioxidant compounds, which translated into the maintenance of redox balance, as indicated by the GSSG/GSH ratio [14,52].

Surprisingly, these effects are like those obtained previously in TH-HZ Swiss mice [10]. However, in WT C57BL/6J mice, there was no sexual dimorphism in the stress response that we observed in the Swiss background.

WT C57BL/6J males, as well as females, presented an adaptive response to stress, resulting in improved immune function and increased levels of oxidative compounds and antioxidant defenses. In Swiss males exposed to stress, these capacities remained the same or deteriorated, which was a different response from that of females, who showed improved responses.

Generally, the effects on WT C57BL/6 mice observed at the behavioral level, such as increased anxiety and a reduced exploratory capacity, could be due to the effect of restraint stress, since it has been observed that preventing the movement of these animals causes them a high level of anxiety [53]. Thus, when this stress is applied, this anxiety behavior could decrease their exploratory capacity, either making them explore less or producing an aberrant exploration derived from the stress to which they have been subjected, which in no case would be a directed exploration [53].

In addition, the CA that regulate the immune system are mainly noradrenaline and dopamine [6,7,54,55,56,57,58]. Therefore, this improvement in immune system function could be caused by the increases in the levels of some of these hormones in response to stress [10]. Finally, the main hormone secreted under stressful conditions is adrenaline, which is directly related to the production of proinflammatory cytokines, and explains, in our case, the increase in basal proliferation observed in WT mice after stress [59].

Moreover, the TH-HZ mice in our study showed an increase in anxiety-like behaviors, as indicated by a greater number of grooming/freezing behaviors in the different tests [60]. In addition, TH-HZ females exhibited impaired innate and adaptive immunity, whereas males only exhibited impaired innate immunity. With respect to the oxidative state, TH-HZ mice did not differ with respect to the basal state, except for males, which presented a relatively high concentration of GSSG.

In the case of TH-HZ mice, a less evident stress response due to sex was observed. TH-HZ C57BL/6J males exhibited more oxidative stress than females, as indicated by the GSSG/GSH ratio [14,52]. These findings are consistent with those reported in a previous study using the Swiss strain [10], but again, in the C57BL/6J strain, the differences due to sex are considerably blunted.

This study reveals several interesting findings. First, it shows the importance of correct CA production in developing an adequate function of homeostatic systems and coping with stress. Second, it reveals how, in the context of CA physiology, the strain employed to develop GEMs is key for the results obtained, making it an essential factor to consider, at least in this framework.

However, the present work was only conducted using a single inbred strain, providing a controlled genetic background that strengthens the consistency of the observed effects. Nevertheless, extending this approach to additional inbred strains will be an important future step to assess how genetic variability may shape these outcomes and to further broaden the significance of our findings. It should also be noted that animals carrying *Th* haploinsufficiency from conception may undergo physiological adaptations during ontogeny; therefore, the direct effects of this genetic alteration could be further clarified using inducible or adult-onset gene suppression models.

Therefore, we can conclude that the TH-HZ mice that were developed in the C57BL/6J strain presented behavioral impairments, immunosenescence, and oxidative stress with respect to the WT mice. After acute restraint stress, while WT mice can develop an adaptive stress response, improving their immune function, TH-HZ mice, given their limited production of CA, do not respond correctly to stress and exhibit deterioration of several nervous and immune functions. Furthermore, the C57BL/6J strain is a very homogeneous strain with less evident sexual dimorphism in terms of behavior, immunity, and the redox state, with the sex-dependent stress response being minimal, at least in the context of *Th* haploinsufficiency.

## 4. Materials and Methods

### 4.1. Animals

In this study, adult (9 ± 1 month) virgin female and male TH-HZ and wild-type (WT) C57BL/6J mice, obtained from different colonies maintained in Dr. Flora de Pablo’s laboratory, were used [61,62]. TH-HZ mice are heterozygous animals for the tyrosine hydroxylase gene, since complete loss of this gene is lethal, and are characterized by a reduction in catecholamine synthesis that, without affecting their overall development or viability, induces early alterations consistent with typical aging processes. For this reason, they have been proposed as a model of premature aging [8,61]. The transgenic mice (TH-HZ) remained healthy and normal, and no signs of any associated lesions were observed. The growth rates were like those of their WT counterparts. Animals were obtained after 20 consecutive generations of parental and offspring mating (all C57BL/6J) to generate an inbred colony. To minimize possible cage-related effects, animals were maintained 6 per cage and separated according to sex and genotype from postnatal day 21. Experimental groups were created after the mothers had weaned the pups to maintain the mice in the same cage from the same litter to avoid hierarchical competition between cage mates. This experimental strategy has broadly been recommended by several authors to avoid or at least minimize these behaviors [63,64]. Mice were provided with ad libitum access to food and tap water and maintained under a reversed 12:12 h light/dark cycle (lights off at 8:00 AM) to minimize circadian influences. Environmental conditions were controlled at 22 ± 2 °C with 50–60% humidity. Animals were fed the A04 diet (Panlab S.L., Barcelona, Spain) in accordance with the American Institute of Nutrition guidelines for laboratory rodents. All experiments were conducted during the dark phase of the light/dark cycle (8:00–12:00 h). Experimental procedures were approved by the Experimental Animal Committee of Complutense University of Madrid (Spain) (PROEX 224.0/21). All mice were handled in accordance with the European Community Council Directive ECC/566/2015, and the study adhered to the ARRIVE guidelines established by the NC3Rs to promote animal welfare and minimize the number of animals used [65].

### 4.2. Experimental Design

**Figure 4:**
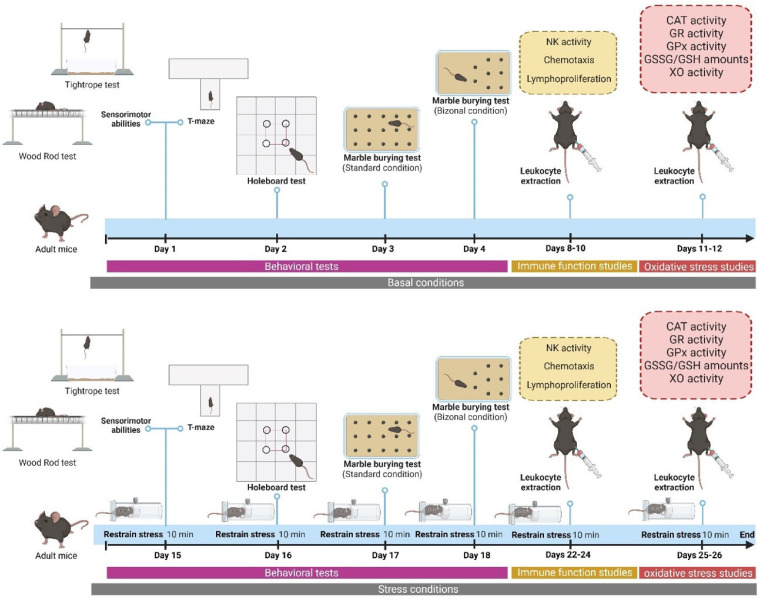
Experimental protocol followed in the work. Male and female WT and TH-HZ C57BL/6J mice were exposed to a battery of behavioral tests as follows: day 1: sensorimotor abilities (visual placing and hindlimb extension reflexes, wood rod test, tightrope test), corner test, and T-maze; day 2: holeboard test; day 3: marble-burying test (standard condition); and day 4: marble-burying test (Bizonal condition). After that, peritoneal suspensions were extracted on days 8, 9, and 10, and several immune functions were assessed in these samples. This peritoneal extraction was repeated on days 11 and 12, and several oxidative stress parameters were analyzed in this last case. This same protocol was performed after applying an acute restraint stress (10 min) to all animals before the trial or peritoneal extraction, as indicated in the figure.

The mice were classified at postnatal day 21 into the following groups to perform this study: female WT (*n* = 6), female TH-HZ (*n* = 6), male WT (*n* = 6), and male TH-HZ mice (*n* = 6). These experimental groups were formed randomly with animals from different litters, meaning that each group is made up of 6 different litters. The sample size (*n* = 6 per group) was established based on previous studies conducted with this model, in which this number of animals was sufficient to detect statistically significant differences between genotypes or sexes. This size was considered to adequately balance the need for statistical rigor with the principle of reducing the use of animals (3R). All the animals were exposed to a behavioral test battery to evaluate their sensorimotor abilities, anxiety-like behaviors, and exploratory capacities. The timeline of the behavioral experiments is shown in Figure 4. Then, peritoneal leukocytes were extracted from all animals, and several immune function and oxidative stress parameters were analyzed using these samples. These studies were considered the basal condition. Animals were exposed to 10 min of restraint stress prior to each analysis and then underwent the same behavioral assessments as well as immune and oxidative stress evaluations under post-stress condition. All cages and sample tubes were coded with opaque alphanumeric codes so that the researchers responsible for functional testing, data collection, and statistical analysis remained blind to the genotype and sex of the animals until the results were processed.

### 4.3. Acute Stress

Animals were individually restrained in 50 mL conical tubes made of polypropylene, equipped with ventilation holes, for 10 min starting at 9 a.m., and this protocol was repeated each day of behavioral testing. Immediately after the restraint protocol was finished, the animals were placed in the behavioral apparatus to study their corresponding behaviors. Additionally, before peritoneal leukocyte extraction to evaluate immune functions and redox parameters, the animals were exposed to restraint stress again.

### 4.4. Behavioral Trials

Consistent with previous studies, behavioral assessments were conducted over four consecutive days [8,66]. On the first day, animals underwent the full battery of sensorimotor assessments: (1) reflexes, (2) corner test, (3) wood rod test, (4) tightrope test, and (5) T-maze. On the second day, mice underwent the holeboard test, followed by 24 h of isolation. On the third and fourth days, they were subjected to the marble-burying test. All behavioral experiments were conducted under red lighting with a 20 W white lamp, positioning the mice in the area of the apparatus considered behaviorally neutral to avoid artificially inducing specific patterns [67]. Between each animal, the equipment was thoroughly wiped with 70% ethanol to minimize olfactory interference. We recorded all the behaviors, indicating the time each was performed, to ensure that specific behaviors, such as grooming, rearing, and freezing, did not depend on the different trial lengths. Afterward, only those behaviors that appeared during all trials were considered. All the behavioral assessments were performed by direct observation by two independent observers who were blinded to the genotype in a counterbalanced manner and with a computerized video system.

#### 4.4.1. Sensorimotor Abilities

##### Visual Placing and Hindlimb Extensor Reflexes

Visual placing and hindlimb extensor reflexes were assessed using a previously described method [68]. Mice were briefly lifted by the tail and positioned onto a black surface for the assessment. A positive response was considered when the forelimbs and hindlimbs of the animals were fully extended. The response was recorded across three trials, and the mean value was calculated.

##### Wood Rod Test

Mice were placed at the center of a wooden rod measuring 2.9 cm in width and 80 cm in length (divided into 10 cm segments) and suspended 22 cm in the air to analyze motor coordination and balance. Two pedestals were employed to secure the wooden rod. Motor coordination was assessed using the latency to leave the starting segment, the number of segments crossed, and the total time spent on the rod. Balance was studied by the time at which the animals completed the trial, the percentage of time that the mice fell, and the latency to fall [68]. Additional behaviors, including the number and latency of freezing episodes and the percentage of animals exhibiting this behavior, were also analyzed.

##### Tightrope Test

This test is widely used to evaluate motor coordination, muscular performance, and traction ability [68]. The apparatus consists of a 60 cm long tightrope, segmented into 10 cm sections, positioned at an elevated height (40 cm) held by two metallic bases. At the start of the behavioral test, each animal was hung by its forelimbs in the middle of the tightrope. Motor coordination was assessed by the total number of segments crossed and the duration spent on the rope (seconds). Muscular strength was evaluated by recording the percentage of mice that fell, the latency to fall (seconds), the proportion of animals completing the trial, and the time required to complete the task (seconds). Finally, motor coordination was evaluated by examining which body parts—forelimbs, hindlimbs, and tail—the animals used to maintain their grip. Responses were categorized to facilitate analysis as low (forelimbs only), medium (forelimbs and hindlimbs), and maximum traction (forelimbs, hindlimbs, and tail).

#### 4.4.2. Exploratory and Anxiety-like Behavioral Tests

##### T-Maze Test

The T-maze test is employed to analyze spontaneous horizontal exploratory behavior [68]. The apparatus consisted of a T-shaped maze with short arms measuring 25 × 10 cm, long arms 65 × 10 cm, and walls 20 cm high. All animals were placed facing the wall in a short arm, considered the most behaviorally neutral area of the maze. The variables analyzed included the time spent at the intersection (seconds) and the total time spent exploring the maze (horizontal exploration). This last parameter refers to the time the animal spent touching the end of each arm. Additionally, vertical exploration was assessed by recording the number of rearing behaviors and their duration (seconds). Grooming and freezing behaviors were also measured, including both frequency and duration.

##### Corner Test

Spontaneous horizontal exploration can also be analyzed by the corner test [8]. For this analysis, a square cage (22 cm) is employed. The variables studied were the number of visited corners, wall rearing behaviors, grooming behaviors, and scratches performed for 30 s.

##### Holeboard Test

The holeboard consists of a 60 × 60 × 45 cm box divided into 36 squares (10 × 10 cm), with four equidistant holes (3.8 cm diameter) located in the central area. This test evaluates non-goal-directed behaviors, measured through horizontal and vertical activity, and goal-directed behaviors, assessed by the number and duration of head-dipping. In this study, the inner zone was defined as the 4 central squares, the outer zone as the 20 squares adjacent to the walls, and the remaining 12 squares as the middle zone. For goal-directed behavior, plastic objects were placed to attract the attention of the mice. The duration of the trial was 5 min [8,68]. Non-goal-directed behavior was quantified as total, outer, middle, and inner locomotion, the mean locomotion per area (number of squares crossed in each zone divided by the total number of squares in that zone), and the percentage of locomotion in each area (number of squares crossed in each zone divided by total locomotion). These measures were considered indicators of horizontal activity. Vertical activity was evaluated by recording the number of wall and central rearing behaviors, as well as the duration of these behaviors (seconds). Goal-directed activity was evaluated by quantifying both the total number of head dippings and the time spent on each dip. Other behaviors (number and time of grooming and freezing behaviors) were also recorded.

##### Marble-Burying Test

The ability of rodents to interact with objects they find dangerous in the environment is reflected by burying behavior [69]. For this experiment, mice were habituated to isolation 24 h prior to testing. Behavioral trials were conducted under standard and bi-zonal conditions. In the standard condition, 12 marbles were placed evenly in the cage, and the mouse was introduced. After 15 min of interaction, the numbers of moved, intact, and buried marbles were recorded. Twenty-four hours after the standard conditions were established, the mice were exposed to the bizonal condition. For the bizonal trial, eight marbles were placed in one section of the cage, leaving the opposite half free. Following 20 min of exposure, moved, intact, and buried marbles were counted.

### 4.5. Extraction of Peritoneal Leukocytes

Peritoneal cell suspensions were collected between 9:00 and 12:00 a.m. to minimize circadian influences. Animals were immobilized by holding the cervical skin, and 3 mL of Hank’s solution at 37 °C was injected into the peritoneal cavity [70]. Following abdominal massage, approximately 80% of Hank’s solution was recovered. Macrophages and lymphocytes were identified and counted using a Neubauer chamber. Cell viability was assessed using the Trypan blue exclusion test (RRID:CHEBI_78897; Sigma-Aldrich, St. Louis, MO, USA), with viability exceeding 98% in all cases. Peritoneal suspensions were then adjusted to specific concentrations of macrophages, lymphocytes, or total leukocytes, depending on the parameter being analyzed, as described in each corresponding test section. A Neubauer chamber was used to adjust the different cell types, and lymphocytes and macrophages were differentiated by their morphology using 40× microscopy.

### 4.6. Immune Function Parameters

#### 4.6.1. Chemotaxis

Peritoneal leukocyte chemotaxis was determined according to a previous study [12]. This chemotaxis assay evaluates the ability of immune cells to migrate toward an infectious focus. Macrophages or lymphocytes (0.5 × 10^6^ cells/mL in Hank’s solution) were added to the upper chamber of a Boyden setup, with the chemoattractant f-Met-Leu-Phe (RRID:CHEBI_16552; Sigma, St. Louis, MO, USA) in the lower chamber. After 3 h, cells on the filters were fixed and stained with Giemsa, and the chemotactic index was determined by counting cells on one-third of the lower face of each filter.

#### 4.6.2. Natural Killer Activity

Natural killer activity was analyzed using a previously reported protocol [12]. A suspension of 10^6^ peritoneal leukocytes/mL in RPMI 1640 medium was plated in 96-well U-bottom plates. Murine YAC-1 lymphoma cells were added to the wells, and NK cell activity was evaluated by measuring lactate dehydrogenase release into the medium (Cytotox 96™, Promega, Walldorf, Germany). Results are expressed as the percentage of lysed tumor cells (% lysis).

#### 4.6.3. Lymphoproliferative Capacity

Basal lymphocyte proliferation (without stimulus) and proliferation in response to concanavalin A (1 µg/mL ConA; RRID:CHEBI_232423; Sigma-Aldrich) or lipopolysaccharide (1 µg/mL LPS, *Escherichia coli* 055:B5; RRID:CHEBI_16412; Sigma-Aldrich) were assessed as previously described [12,70]. For this purpose, 10^6^ lymphocytes/mL in RPMI 1640 medium supplemented with gentamicin (RRID:CHEBI_16264) and fetal bovine serum (FBS) were added to 96-well plates. Following a 48-h incubation at 37 °C in a sterile, humidified 5% CO_2_ atmosphere, 3H-thymidine (RRID:CHEBI_16717) was added to the cultures and incubated for an additional 24 h. Cells were then harvested using a semi-automatic harvester, and 3H-thymidine incorporation was measured with a beta counter. Results are expressed as counts per minute (c.p.m.).

### 4.7. Parameters of Oxidative Stress

#### 4.7.1. Catalase Activity

Catalase activity (RRID:CHEBI_16475) was evaluated by centrifuging 10^6^ leukocytes/mL in Hank’s solution for 5 min at 1500 rpm. Cell pellets were resuspended in 50 mM oxygen-free phosphate buffer and sonicated. The resulting supernatants were then used in an enzymatic reaction with 14 mM H_2_O_2_ (RRID:CHEBI_15379) as the substrate. The enzymatic assay was performed spectrophotometrically for 80 s at 240 nm by measuring the decomposition of H_2_O_2_ into H_2_O + O_2_, as previously described [8]. Results are expressed as international units (IUs) of enzymatic activity per 10^6^ peritoneal leukocytes.

#### 4.7.2. Glutathione Reductase Activity

Glutathione reductase activity (RRID:CHEBI_16736) was assessed using 10^6^ leukocytes/mL in Hank’s solution. Briefly, cells were centrifuged at 1500 rpm for 5 min, and the pellets were resuspended in 50 mM oxygen-free phosphate buffer containing 6.3 mM EDTA (RRID:CHEBI_27497). Cells were subsequently sonicated, and the supernatants were incubated in an enzymatic reaction using 80 mM GSSG as the substrate. NADPH oxidation (RRID:CHEBI_16476) was monitored spectrophotometrically by measuring the decrease in absorbance at 340 nm over 240 s [8]. Results are expressed as milliunits (mU) of enzymatic activity per 10^6^ peritoneal leukocytes.

#### 4.7.3. Glutathione Peroxidase Activity

Cell suspensions adjusted to 10^6^ leukocytes/mL in Hank’s solution were centrifuged at 1500 rpm for 5 min. Pelleted cells were resuspended in 50 mM oxygen-free phosphate buffer and sonicated. The supernatants were incubated in an enzymatic reaction using cumene hydroperoxide (cumene-OOH; RRID:CHEBI_33453) as substrate. NADPH oxidation (RRID:CHEBI_16476) was monitored spectrophotometrically by recording the decrease in absorbance at 340 nm over 300 s [8]. The results are presented as mUs of enzymatic activity per 10^6^ peritoneal leukocytes.

#### 4.7.4. Glutathione Concentrations

A total of 10^6^ leukocytes/mL in Hank’s solution were used to analyze glutathione concentrations (RRID:CHEBI_29985). The cell pellets were then resuspended in 50 mM phosphate buffer (pH 8) containing 0.1 mM EDTA (RRID:CHEBI_27497) and sonicated. Afterward, the supernatants were collected and used to quantify reduced (GSH; RRID:CHEBI_57945) and oxidized (GSSG; RRID:CHEBI_29986) glutathione. This protocol is based on the reaction capacity of GSSG and GSH in the presence of o-phthalaldehyde (OPT; RRID:CHEBI_16752) at pH 12 and pH 8, respectively. A fluorescent product was generated and its intensity was recorded using 350 nm excitation and 420 nm emission. [8]. The results are presented as nmol of GSSG and GSH per 10^6^ peritoneal leukocytes. The GSSG/GSH ratio was also reported.

#### 4.7.5. Xanthine Oxidase Activity

Xanthine oxidase (XO; RRID:CHEBI_15893) activity was determined using the commercial A-22182 Amplex Red Xanthine/Xanthine Oxidase Assay Kit (Molecular Probes, Paisley, UK). Leukocytes were prepared at 10^6^ cells/mL in Hank’s solution. Cell pellets were resuspended in 50 mM potassium phosphate buffer containing 0.1 M EDTA (RRID:CHEBI_27497) and 0.5 mM DTT (RRID:CHEBI_58094). The supernatants were incubated with the Amplex Red working solution, and fluorescence was recorded at 530 nm excitation and 595 nm emission. Results are expressed as units (U) of enzymatic activity per 10^6^ peritoneal leukocytes.

### 4.8. Statistical Analysis

Statistical analyses were conducted using GraphPad Prism 9.5.1 (GraphPad Software, LLC, San Diego, CA, USA). Data are expressed as mean ± standard deviation (SD). Comparisons between the groups were analyzed using repeated-measures ANOVA. The independent factors were genotype and sex, with measurements taken at two time points, basal and post-stress. The analysis evaluated the interactions between these factors and their main effects on the dependent variable. The Greenhouse–Geisser correction was applied when the sphericity assumption was not met, and post hoc comparisons were conducted using Tukey’s test. Differences were considered statistically significant at *p* < 0.05.

## Figures and Tables

**Table 1:** Sensorimotor abilities in female and male WT and TH-HZ mice under basal and post-stress conditions.

	Basal				Post-Stress			
	Females		Males		Females		Males	
	WT	TH-HZ	WT	TH-HZ	WT	TH-HZ	WT	TH-HZ
**Wood Rod Test**								
Number of freezings	0	3 ± 1 aaa	0	0 ###	0	9 ± 2 aaa***	0	0 ###
Time of freezing (s)	0	7 ± 2 aaa	0	0 ###	0	17 ± 3 aaa***	0	0 ###

Values are expressed as mean ± standard deviation (*n* = 6). aaa *p* < 0.001 compared to WT. ### *p* < 0.001 compared to female. *** *p* < 0.001 compared to basal condition.

**Table 2:** Assessment of exploratory and anxiety-like behaviors in female and male WT and TH-HZ mice under basal and post-stress conditions.

	Basal				Post-Stress			
	Females		Males		Females		Males	
	WT	TH-HZ	WT	TH-HZ	WT	TH-HZ	WT	TH-HZ
**Holeboard test**								
Non-goal-directed behavior								
Vertical exploration								
Number of central rearings	4 ± 1	0 aaa	4 ± 2	0 aaa	0 ***	0	0 ***	0
Time of central rearings (s)	9 ± 3	0 aaa	10 ± 3	0 aaa	0 ***	0	0 ***	0
Horizontal exploration								
% Central locomotion	41 ± 3	9 ± 2 aaa	40 ± 3	41 ± 3 ###	29 ± 2 ***	40 ± 5 aaa***	38 ± 5 ##	24 ± 6 aaa###***
% Peripherical locomotion	59 ± 9	92 ± 3 aaa	60 ± 5	59 ± 4 ###	61 ± 4	60 ± 9 ***	62 ± 5	76 ± 10 a##**
Other behaviors								
Number of groomings	0	6 ± 1 aaa	0	5 ± 2 aa	8 ± 3 ***	15 ± 3 aaa***	3 ± 1 ##	12 ± 3 aaa***
Time of groomings (s)	0	10 ± 2 aaa	0	13 ± 3 aaa	14 ± 5 ***	21 ± 2 aaa***	7 ± 2 ###***	17 ± 2 aaa
Number of freezings	0	3 ± 1 a	0	0 #	3 ± 2 *	10 ± 2 aaa***	2 ± 1	6 ± 3 aa##***
Time of freezings (s)	0	7 ± 3 aaa	0	0 ###	5 ± 2 **	14 ± 4 aaa***	5 ± 1 **	13 ± 3 aaa***
Goal-directed behavior								
Number of head dippings	10 ± 3	4 ± 2 aa	15 ± 2 #	5 ± 1 aaa	13 ± 3	17 ± 3 ***	14 ± 3	4 ± 1 aaa###
Time of head dippings (s)	49 ± 13	12 ± 9 aaa	39 ± 3 #	14 ± 3 aaa	4 ± 2 ***	5 ± 2	16 ± 2 ##***	6 ± 2 a#
**T-Maze test**								
Horizontal exploration								
Exploratory efficacy (s)	32 ± 12	43 ± 18	44 ± 12	43 ± 13	25 ± 8	68 ± 12 aaa	31 ± 10	71 ± 24 aaa*
Other behaviors								
Number of groomings	0	1 ± 1	0	0	1 ± 1	4 ± 1 aaa***	1 ± 0	3 ± 1 aaa***
Time of groomings (s)	0	7 ± 2 aaa	0	0 ###	2 ± 1	12 ± 2 aaa***	1 ± 0	5 ± 3 aaa###***
Number of freezings	0	0	0	0	0	5 ± 1 aaa***	0	0 ###
Time of freezings (s)	0	0	0	0	0	5 ± 1 aaa***	0	0 ###
**Burial behavior**								
Standard condition								
Number of intact pieces	10 ± 2	6 ± 1 aa	10 ± 1	4 ± 2 aaa	10 ± 2	5 ± 1 aaa	10 ± 2	3 ± 1 aaa
Number of moved pieces	1 ± 1	5 ± 2 aaa	2 ± 1	8 ± 2 ##	2 ± 1	7 ± 1 aaa	1 ± 1	4 ± 1 ###
Number of buried pieces	1 ± 1	3 ± 1	1 ± 1	4 ± 2 aa	2 ± 1	4 ± 1	1 ± 1	5 ± 1 aaa
Bizonal condition								
Number of intact pieces	7 ± 1	2 ± 1 aaa	5 ± 1	2 ± 1 aa	8 ± 1	4 ± 1 aaa	5 ± 1 ##	3 ± 2
Number of moved pieces	1 ± 1	5 ± 2 aaa	3 ± 2	6 ± 2 a	1 ± 1	4 ± 1 a	2 ± 1	4 ± 1
Number of buried pieces	1 ± 1	5 ± 1 aaa	3 ± 2	4 ± 2	0	3 ± 1 aa	2 ± 1	4 ± 1

Values are expressed as mean ± standard deviation (*n* = 6). a *p* < 0.05, aa *p* < 0.01, aaa *p* < 0.001 compared to WT. # *p* < 0.05, ## *p* < 0.01, ### *p* < 0.001 compared to female. * *p* < 0.05, ** *p* < 0.01, *** *p* < 0.001 compared to basal condition.

**Table 3:** Assessment of immune function in peritoneal leukocytes of female and male WT and TH-HZ mice under basal and post-stress conditions.

	Basal				Post-Stress			
	Females		Males		Females		Males	
	WT	TH-HZ	WT	TH-HZ	WT	TH-HZ	WT	TH-HZ
**Macrophage functions**								
Chemotaxis index (C.I)	398 ± 58	293 ± 27 aa	387 ± 65	265 ± 49 aa	531 ± 40 ***	174 ± 32 aaa**	654 ± 54 ##***	197 ± 39 aaa
**Lymphocyte functions**								
Chemotaxis index (C.I)	489 ± 29	354 ± 48 aaa	532 ± 47	408 ± 29 aaa	581 ± 39 *	197 ± 54 aaa***	498 ± 74	116 ± 32 aaa***
Natural killer activity (%)	23 ± 3	27 ± 3	18 ± 3	24 ± 2 aa	35 ± 2 ***	19 ± 2 aaa***	15 ± 4 ###	9 ± 2 aa###***
Lymphoproliferation								
Basal proliferative response (c.p.m)	1009 ± 222	1802 ± 381 aaa	1031 ± 164	2298 ± 239 aaa	2632 ± 322 ***	1580 ± 161 aaa	2354 ± 322 ***	1654 ± 290 aa**
Proliferative response to LPS (c.p.m)	2546 ± 299	1273 ± 101aaa	2435 ± 254	1277 ± 233 aaa	3009 ± 129 *	1045 ± 204 aaa	3321 ± 157 ***	1119 ± 289 aaa
Proliferative response to ConA (c.p.m)	2354 ± 322	1486 ± 188 aaa	2214 ± 322	1186 ± 291 aaa	3207 ± 201 ***	1032 ± 177 aaa	3985 ± 299 ###***	1298 ± 392 aaa

Values are expressed as mean ± standard deviation (*n* = 6). aa *p* < 0.01, aaa *p* < 0.001 compared to WT. ## *p* < 0.01, ### *p* < 0.001 compared to female. * *p* < 0.05, ** *p* < 0.01, *** *p* < 0.001 compared to basal condition.

**Table 4:** Assessment of oxidative stress parameters in peritoneal leukocytes of female and male WT and TH-HZ mice under basal and post-stress conditions.

	Basal				Post-Stress			
	Females		Males		Females		Males	
	WT	TH-HZ	WT	TH-HZ	WT	TH-HZ	WT	TH-HZ
**Antioxidant compounds**								
Catalase activity (UI CAT/10^6^ cells)	7 ± 2	4 ± 1	5 ± 2	3 ± 1	14 ± 4 ***	5 ± 1 aaa	8 ± 2 ###	6 ± 3 ###
Glutathione reductase activity (mU GR/10^6^ cells)	36 ± 7	32 ± 12	44 ± 15	38 ± 13	42 ± 3	31 ± 2	67 ± 10 ##**	27 ± 9 aaa
Glutathione peroxidase activity (mU GPx/10^6^ cells)	149 ± 30	216 ± 39	167 ± 38	226 ± 82	375 ± 37 ***	221 ± 39 aaa	431 ± 32 ***	220 ± 38 aaa
Reduced glutathione levels (GSH) (nmol GSH/10^6^ cells)	39 ± 4	15 ± 4 aaa	29 ± 2 ###	9 ± 5 aaa	64 ± 3 ***	16 ± 2 aaa	54 ± 4 ###***	12 ± 4 aaa
**Oxidant compounds**								
Xanthine oxidase activity (U XAO/10^6^ cells)	0.58 ± 0.20	0.84 ± 0.15	0.80 ± 0.12	1.41 ± 0.39	2.54 ± 0.33 ***	1.96 ± 0.72 ***	3.04 ± 0.43 ***	2.74 ± 0.78 aaa###***
Oxidized glutathione levels (GSSG) (nmol GSSG/10^6^ cells)	1.32 ± 0.07	2.5 ±0.09 aaa	1.75 ± 0.03 ###	2.25 ± 0.18 aaa#	2.31 ± 0.02 ***	1.96 ± 0.13 aaa***	2.74 ± 0.02 ###***	3.06 ± 0.25 aa###***
**Redox state indicator**								
GSSG/GSH ratio	0.03 ± 0.01	0.14 ± 0.03 aaa	0.06 ± 0.01	0.25 ± 0.08 aaa###	0.04 ± 0.01	0.12 ± 0.01 aa	0.05 ± 0.02	0.26 ± 0.01 aaa###

Values are expressed as mean ± standard deviation (*n* = 6). aa *p* < 0.01, aaa *p* < 0.001 compared to WT. # *p* < 0.05, ## *p* < 0.01, ### *p* < 0.001 compared to female. ** *p* < 0.01, *** *p* < 0.001 compared to basal condition.

## Data Availability

Data will be available upon request to Judith Félix (jufelix@ucm.es) or Antonio Garrido (antonio.garrido@universidadeuropea.es).

## Supplementary Materials

The following supporting information can be downloaded at: https://www.mdpi.com/article/10.3390/ijms26188818/s1.
