# Supplementary material for: Haploinsufficiency of the Tyrosine Hydroxylase Gene in the Inbred C57BL/6J Strain Alters Behavior, Immunity, and Oxidative Stress, Especially After Acute Stress
Source: Int J Mol Sci. 2025 Sep 10;26(18):8818. doi: 10.3390/ijms26188818 (PMC12469802; doi:10.3390/ijms26188818)
Supplement: Supplementary file 1 [file ijms-26-08818-s001.zip › ijms-3776279-supplementary.pdf]

Supplementary Table S1. Comparison between strain and sex of the different parameters evaluated in TH-HZ mice respect WT mice before and after an acute stress.

	**Basal**				**Post-stress**			
	**Females**		**Males**		**Females**		**Males**	
	**Swiss**	**C57BL/6J**	**Swiss**	**C57BL/6J**	**Swiss**	**C57BL/6J**	**Swiss**	**C57BL/6J**
**Tightrope test**								
Muscular vigor								
Latency to fall (s)	↓↓↓	↑	↑↑↑	↑↑	=	=	=	=
**Holeboard test**								
Exploration and anxiety								
Inner locomotion	↓↓↓	↓↓↓	↓↓	↓↓↓	↓↓	=	↓↓	↓
Time of groomings (s)	↑↑↑	↑↑↑	↑	↑↑↑	=	↑↑	↑	=
Goal-directed exploration								
Time of Head-dipping (s)	↓↓↓	↓↓↓	↓↓	↓↓↓	↓↓↓	=	↓↓	=
**Macrophage functions**								
Chemotaxis index (C.I)	↓↓↓	↓	↓↓↓	↓↓	↓↓↓	↓↓↓	=	=
**Lymphocyte functions**								
Chemotaxis index (C.I)	↓↓↓	↓↓	↓↓↓	↓↓↓	↓↓↓	↓↓↓	↓	↓↓↓
Natural Killer activity (%)	↓↓↓	=	↓↓↓	=	=	↓↓	↓↓	↓↓↓
Lymphoproliferation								
Basal proliferative response (c.p.m)	↑↑↑	↑↑	↑↑↑	↑↑↑	=	=	↓↓	↓↓
Proliferative response to LPS (c.p.m)	↓↓↓	↓↓↓	↓↓↓	↓↓↓	↓↓↓	=	↓↓↓	=
Proliferative response to ConA (c.p.m)	=	↓↓↓	↑	↓↓↓	↓↓↓	↓	↓↓↓	=
**Antioxidant compounds**								
Catalase activity (IU CAT/10^6 cells)	↓↓↓	=	=	=	=	=	=	=
Glutathione reductase activity (mU GR/10^6 cells)	↓	=	↓↓	=	↑	=	↑↑↑	=
Glutathione peroxidase activity (mU GPx/10^6 cells)	=	↑	↓	↑↑	=	=	=	=
Reduced glutathione levels (GSH) (nmol GSH/10^6 cells)	↓↓↓	↓↓↓	↓↓	↓↓↓	=	=	=	=
**Oxidant compounds**								
Xanthine oxidase activity (U XO/10^6 cells)	↑↑↑	=	=	↓	↑↑↑	=	=	=
Oxidized glutathione levels (GSSG) (nmol GSSG/10^6 cells)	↑	↑↑↑	↑↑↑	↑↑	↑↑	=	↑↑↑	↑↑↑
**Redox state indicator**								
GSSG/GSH Ratio	↑↑↑	↑↑↑	↑↑	↑↑↑	↑↑↑	=	↑	=

↓ p<0,05; ↓↓ p<0,01 and ↓↓↓ p<0.001 decrease; as well as ↑ p<0.05; ↑↑ p<0.01; ↑↑↑ p<0.001 increase, compared to the values obtained in their corresponding control. Swiss strain data were obtained from [10].

Supplementary Table S2. Sensorimotor abilities evaluated in female and male WT and TH-HZ in basal and post-stress conditions.

	Basal				Post-stress			
	Females		Males		Females		Males	
	WT	TH-HZ	WT	TH-HZ	WT	TH-HZ	WT	TH-HZ
**Weight (g)**	25±1	26±2	30±1 ###	29±2 #	25±2	25±2	29±1 ##	31±1###
**Visual placing reflex**								
Mice showing this response	6/6	6/6	6/6	6/6	6/6	6/6	6/6	6/6
**Hindlimb extensor reflex**								
Mice showing this response	6/6	6/6	6/6	6/6	6/6	6/6	6/6	6/6
**Wood rod test**								
Motor coordination								
Total number of crossing segments	4±2	5±2	4±1	4±0	5±2	4±2	4±2	4±1
Time of permanence (s)	41±23	32±25	21±13	17±10	32±20	23±17	24±16	24±23
Balance								
Mice falling off the wood rod	0/6	0/6	0/6	0/6	0/6	0/6	0/6	0/6
Latency to fall (s)	0	0	0	0	0	0	0	0
Mice complete the test	4/6	4/6	6/6	6/6	5/6	5/6	5/6	5/6
Time to complete the test (s)	36±24	31±25	21±13	17±10	28±17	18±10	19±7	11±3
Freezing behavior								
% Mice performing freezing	0/6	4/6	0/6	0/6	0/6	6/6	0/6	0/6
**Tightrope test**								
Motor coordination								
Total number of crossing segments	3±2	3±2	4±1	3±2	2±2	2±2	4±1	3±1
Time of permanence (s)	33±18	37±16	39±14	36±17	32±19	26±20	31±15	35±18
Muscular vigor								
Mice completing the test	6/6	4/6	6/6	5/6	2/6 **	2/6 *	5/6 #	4/6 #
Mice falling off the tightrope	0/6	1/6	0/6	1/6	2/6	2/6	1/6	1/6
Latency to fall (s)	-	9±3 aa	-	20±6 aaa###	23±4 ***	9±5 aaa	48±0 ###***	17±5 aaa#
Time to complete the test (s)	33±15	33±13	33±8	27±8	28±6	35±12	28±14	41±17
Traction								
Low	0/6	1/6	0/6	1/6	1/6	3/6	0/6	2/6 a
Medium	0/6	1/6	0/6	0/6	1/6	1/6	0/6	1/6
Maximum	6/6	4/6	6/6	5/6	4/6	2/6 a*	6/6	3/6 a*

Each value represents the mean ± standard deviation of values corresponding to 6 animals. a $p<0.05$, aa $p<0.01$, aaa $p<0.001$ compared to WT. # $p<0.05$, ## $p<0.01$, ### $p<0.001$ compared to female. *** $p<0.001$ compared to basal condition.

Supplementary Table S3. Exploratory and anxiety-like behaviors evaluated in female and male WT and TH-HZ in basal and post-stress conditions.

	Basal				Post-stress			
	Females		Males		Females		Males	
	WT	TH-HZ	WT	TH-HZ	WT	TH-HZ	WT	TH-HZ
**Holeboard test**								
Non goal directed behavior								
Vertical exploration								
Number of wall rearings	10±2	15±6	8±2	14±4	15±3	20±3	9±3	18±3 aa
Time of wall rearings (s)	18±4	23±6	14±2	20±5	21±5	22±3	16±4	23±2
Horizontal exploration								
Central locomotion	60±3	11±2 aaa	103±3 ###	68±4 aaa###	91±7 ***	78±11***	63±11 ***###	27±10 aaa###***
Peripherical locomotion	84±6	121±13aaa	154±15 ###	98±13 aaa#	144±15 ***	118±6 a	100±12 ###***	77±11 a###
Total locomotion	144±13	132±24	257±25 ###	166±31 aaa	235±22 ***	196±24 **	163±32 ###***	104±27 aa###
Central locomotion average	4±1	1±1 aaa	6±1 #	4±1 a###	6±1 *	5±1 ***	4±1 #*	2±1 a###*
Peripherical locomotion average	4±1	5±1	8±1 ###	5±1 aa	7±1 **	6±1	5±1 **	4±2
Total locomotion average	4±1	3±1	7±1 ###	5±1 a#	7±1 ***	5±1 a*	5±1 #*	3±1 a#*
**T-Maze test**								
Horizontal exploration								
Intersection time (s)	13±6	12±7	15±5	15±4	7±4	15±3	11±2	24±12 a
Vertical exploration								
Number of rearings	1±1	1±1	1±1	2±1	1±1	0	2±1	0 aa**
Time of rearings (s)	1±1	1±1	3±1 #	3±1 #	1±1	0	3±1 #	0 ###
**Corner test**								
Number of corners	8±1	5±1 a	8±2	4±1 aaa	5±1 *	4±1	6±2	3±2 a
Number of wall rearings	5±1	5±1	6±1	5±1	4±2	3±2	4±1	2±1 **
Number of groomings	0	1±1	1±1	1±1	4±1 ***	7±1 aaa***	2±1 #	3±1 ###*
Number of scratches	1±1	1±1	0	0	1±1	1±1	1±1	0

Each value represents the mean ± standard deviation of values corresponding to 6 animals. a $p<0.05$, aa $p<0.01$, aaa $p<0.001$ compared to WT. # $p<0.05$, ### $p<0.001$ compared to female. * $p<0.05$, ** $p<0.01$, *** $p<0.001$ compared to basal condition.

Supplementary Table S4. Repeated measures ANOVA analysis

	Genotype	Sex	Stress	Genotype x Sex	Genotype x Stress	Sex x Stress	Genotype x Sex x Stress
**Weight (g)**	F(1,20) = 1.2<br>p = 0.28	F(1,20) = 97.2<br>p < 0.001	F(1,20) = 0<br>p > 0.99	F(1,20) = 0<br>p > 0.99	F(1,20) = 1.2<br>p = 0.28	F(1,20) = 1.2<br>p = 0.28	F(1,20) = 4.8<br>p = 0.034
**Behavioral parameters**							
**Wood rod test**							
Motor coordination							
Total number of crossing segments	F(1,20) = 0<br>p > 0.99	F(1,20) = 1.09<br>p = 0.30	F(1,20) = 0<br>p > 0.99	F(1,20) = 0<br>p > 0.99	F(1,20) = 1.09<br>p = 0.30	F(1,20) = 0<br>p > 0.99	F(1,20) = 1.09<br>p = 0.30
Time of permanence (s)	F(1,20) = 1.002<br>p = 0.32	F(1,20) = 3.65<br>p = 0.06	F(1,20) = 0.13<br>p = 0.71	F(1,20) = 0.41<br>p = 0.53	F(1,20) = 0.03<br>p = 0.85	F(1,20) = 1.62<br>p = 0.21	F(1,20) = 0.03<br>p = 0.86
Balance							
Latency to fall (s)	F(1,20) = 0<br>p > 0.99	F(1,20) = 0<br>p > 0.99	F(1,20) = 0<br>p > 0.99	F(1,20) = 0<br>p > 0.99	F(1,20) = 0<br>p > 0.99	F(1,20) = 0<br>p > 0.99	F(1,20) = 0<br>p > 0.99
Time to complete the test (s)	F(1,20) = 2.28<br>p = 0.14	F(1,20) = 6.33<br>p = 0.02	F(1,20) = 2.63<br>p = 0.11	F(1,20) = 0.03<br>p = 0.87	F(1,20) = 0.25<br>p = 0.62	F(1,20) = 0.53<br>p = 0.47	F(1,20) = 0.003<br>p = 0.95
Other behaviors							
Number of freezings	F(1,20) = 0<br>p > 0.99	F(1,20) = 0<br>p > 0.99	F(1,20) = 43.2<br>p < 0.001	F(1,20) = 172.8<br>p < 0.001	F(1,20) = 43.2<br>p < 0.001	F(1,20) = 43.2<br>p < 0.001	F(1,20) = 43.2<br>p < 0.001
Time of freezing (s)	F(1,20) = 265.8<br>p < 0.001	F(1,20) = 265.8<br>p < 0.001	F(1,20) = 46.15<br>p < 0.001	F(1,20) = 265.8<br>p < 0.001	F(1,20) = 46.15<br>p < 0.001	F(1,20) = 46.15<br>p < 0.001	F(1,20) = 46.15<br>p < 0.001
**Tightrope test**							
Motor coordination							
Total number of crossing segments	F(1,20) = 1.04<br>p = 0.31	F(1,20) = 4.17<br>p = 0.05	F(1,20) = 1.04<br>p = 0.31	F(1,20) = 1.04<br>p = 0.31	F(1,20) = 0<br>p > 0.99	F(1,20) = 1.04<br>p = 0.31	F(1,20) = 0<br>p > 0.99
Time of permanence (s)	F(1,20) = 0.002<br>p = 0.96	F(1,20) = 0.43<br>p = 0.52	F(1,20) = 1.11<br>p = 0.29	F(1,20) = 0.02<br>p = 0.88	F(1,20) = 0.02<br>p = 0.88	F(1,20) = 0.02<br>p = 0.88	F(1,20) = 0.73<br>p = 0.39
Muscular vigor							
Latency to fall (s)	F(1,20) = 13.84<br>p < 0.001	F(1,20) = 104.6<br>p < 0.001	F(1,20) = 249.9<br>p < 0.001	F(1,20) = 1.95<br>p = 0.17	F(1,20) = 296<br>p < 0.001	F(1,20) = 26.16<br>p < 0.001	F(1,20) = 42.38<br>p < 0.001
Time to complete the test (s)	F(1,20) = 0.99<br>p = 0.33	F(1,20) = 0<br>p > 0.99	F(1,20) = 0.18<br>p = 0.67	F(1,20) = 0<br>p > 0.99	F(1,20) = 3.42<br>p = 0.07	F(1,20) = 0.73<br>p = 0.39	F(1,20) = 0.73<br>p = 0.39
**Holeboard test**							
Non goal directed behavior							
Vertical exploration							
Number of wall rearings	F(1,20) = 39.06<br>p < 0.001	F(1,20) = 7.56<br>p = 0.01	F(1,20) = 14.06<br>p < 0.001	F(1,20) = 1.56<br>p = 0.22	F(1,20) = 0.56<br>p = 0.46	F(1,20) = 1.56<br>p = 0.22	F(1,20) = 0.56<br>p = 0.45
Time of wall rearings (s)	F(1,20) = 16.04<br>p < 0.001	F(1,20) = 5.38<br>p = 0.03	F(1,20) = 2.18<br>p = 0.15	F(1,20) =2.18<br>p = 0.15	F(1,20) = 0.4<br>p = 0.53	F(1,20) = 0.4<br>p = 0.53	F(1,20) = 1.11<br>p = 0.29
Number of central rearings	F(1,20) = 76.8<br>p < 0.001	F(1,20) = 0<br>p > 0.99	F(1,20) = 76.8<br>p < 0.001	F(1,20) = 0<br>p > 0.99	F(1,20) = 76.8<br>p < 0.001	F(1,20) = 0<br>p > 0.99	F(1,20) = 0<br>p > 0.99
Time of central rearings (s)	F(1,20) = 120.3<br>p < 0.001	F(1,20) = 0.33<br>p = 0.57	F(1,20) = 120.3<br>p < 0.001	F(1,20) = 0.33<br>p = 0.57	F(1,20) = 120.3<br>p < 0.001	F(1,20) = 0.33<br>p = 0.57	F(1,20) = 0.33<br>p = 0.57
Horizontal exploration							
Central locomotion	F(1,20) = 247.4<br>p < 0.001	F(1,20) = 6.17<br>p = 0.02	F(1,20) = 4.04<br>p = 0.05	F(1,20) = 1.13<br>p = 0.29	F(1,20) = 17.13<br>p < 0.001	F(1,20) = 448.1<br>p < 0.001	F(1,20) = 19.15<br>p < 0.001
Peripherical locomotion	F(1,20) = 24.66<br>p < 0.001	F(1,20) = 7.7<br>p = 0.01	F(1,20) = 1.73<br>p = 0.19	F(1,20) = 43.2<br>p < 0.001	F(1,20) = 4.8<br>p = 0.03	F(1,20) = 92.93<br>p < 0.001	F(1,20) = 49.15<br>p < 0.001
Total locomotion	F(1,20) = 47.12<br>p < 0.001	F(1,20) = 0.34<br>p = 0.56	F(1,20) = 0.001<br>p =0.97	F(1,20) = 11.43<br>p = 0.002	F(1,20) = 0.03<br>p = 0.86	F(1,20) = 112.8<br>p < 0.001	F(1,20) = 4.06<br>p = 0.05
Central locomotion average	F(1,20) = 60.75<br>p < 0.001	F(1,20) = 18.75<br>p < 0.001	F(1,20) = 0.75<br>p = 0.39	F(1,20) = 18.75<br>p < 0.001	F(1,20) = 0.75<br>p = 0.39	F(1,20) = 18.75<br>p < 0.001	F(1,20) = 6.75<br>p = 0.01
Peripherical locomotion average	F(1,20) = 8.73<br>p = 0.005	F(1,20) = 0<br>p > 0.99	F(1,20) = 0<br>p > 0.99	F(1,20) = 8.72<br>p = 0.005	F(1,20) = 0<br>p > 0.99	F(1,20) = 34.91<br>p < 0.001	F(1,20) = 8.72<br>p = 0.005
Total locomotion average	F(1,20) = 36.75<br>p < 0.001	F(1,20) = 0.75<br>p = 0.39	F(1,20) = 0.75<br>p = 0.39	F(1,20) = 0.75<br>p = 0.39	F(1,20) = 0.75<br>p = 0.39	F(1,20) = 60.75<br>p < 0.001	F(1,20) = 0.75<br>p = 0.39
% Central locomotion	F(1,20) = 57.32<br>p < 0.001	F(1,20) = 28.56<br>p < 0.001	F(1,20) = 0<br>p > 0.99	F(1,20) = 3.17<br>p = 0.08	F(1,20) = 38.88<br>p < 0.001	F(1,20) = 71.6<br>p < 0.001	F(1,20) = 166.8<br>p < 0.001

% Peripherical locomotion	F(1,20) = 34.42 p < 0.001	F(1,20) = 3.82 p = 0.06	F(1,20) = 2.06 p = 0.16	F(1,20) = 6.14 p = 0.02	F(1,20) = 6.14 p = 0.02	F(1,20) = 40.81 p < 0.001	F(1,20) = 40.81 p < 0.001
Other behaviors							
Number of groomings	F(1,20) = 132.5 p < 0.001	F(1,20) = 14.73 p < 0.001	F(1,20) = 132.5 p < 0.001	F(1,20) = 0.18 p = 0.67	F(1,20) = 4.55 p = 0.04	F(1,20) = 8.91 p = 0.004	F(1,20) = 1.64 p = 0.21
Time of groomings (s)	F(1,20) = 192 p < 0.001	F(1,20) = 7.68 p < 0.001	F(1,20) = 155.5 p < 0.001	F(1,20) = 4.32 p = 0.04	F(1,20) = 4.32 p = 0.04	F(1,20) = 23.52 p < 0.001	F(1,20) = 0 p > 0.99
Number of freezings	F(1,20) = 61.89 p < 0.001	F(1,20) = 20.21 p < 0.001	F(1,20) = 102.3 p < 0.001	F(1,20) = 11.37 p = 0.002	F(1,20) = 20.21 p < 0.001	F(1,20) = 1.26 p = 0.26	F(1,20) = 0 p > 0.99
Time of freezings (s)	F(1,20) = 88.62 p < 0.001	F(1,20) = 9.85 p = 0.003	F(1,20) = 138.5 p < 0.001	F(1,20) = 9.85 p = 0.003	F(1,20) = 15.38 p < 0.001	F(1,20) = 5.54 p = 0.02	F(1,20) = 5.54 p = 0.02
Goal directed behavior							
Number of head dippings	F(1,20) = 63.13 p < 0.001	F(1,20) = 4.69 p = 0.04	F(1,20) = 25.57 p < 0.001	F(1,20) = 42.26 p < 0.001	F(1,20) = 13.04 p = 0.001	F(1,20) = 42.26 p < 0.001	F(1,20) = 13.04 p = 0.001
Time of head dippings (s)	F(1,20) = 180.9 p < 0.001	F(1,20) = 4.89 p = 0.03	F(1,20) = 163 p < 0.001	F(1,20) = 2.35 p = 0.13	F(1,20) = 107.9 p < 0.001	F(1,20) = 4.89 p = 0.03	F(1,20) = 27.86 p < 0.001
**T-Maze test**							
Horizontal exploration							
Intersection time (s)	F(1,20) = 8.03 p = 0.01	F(1,20) = 6.5 p = 0.01	F(1,20) = 0.08 p = 0.77	F(1,20) = 0.72 p = 0.4	F(1,20) = 9.71 p = 0.003	F(1,20) = 1.28 p = 0.26	F(1,20) = 0.32 p = 0.57
Exploratory efficacy (s)	F(1,20) = 31.17 p < 0.001	F(1,20) = 1.59 p = 0.21	F(1,20) = 3.92 p = 0.05	F(1,20) = 0.81 p = 0.37	F(1,20) = 19.2 p < 0.001	F(1,20) = 0.03 p = 0.85	F(1,20) = 0.29 p = 0.59
Vertical exploration							
Number of rearings	F(1,20) = 4 p = 0.05	F(1,20) = 4 p = 0.05	F(1,20) = 4 p = 0.05	F(1,20) = 0 p > 0.99	F(1,20) = 16 p < 0.001	F(1,20) = 0 p > 0.99	F(1,20) = 4 p = 0.05
Time of rearings (s)	F(1,20) = 0.86 p = 0.36	F(1,20) = 69.43 p < 0.001	F(1,20) = 0.86 p = 0.36	F(1,20) = 0.86 p = 0.36	F(1,20) = 0.86 p = 0.36	F(1,20) = 0.86 p = 0.36	F(1,20) = 0.86 p = 0.36
Other behaviors							
Number of groomings	F(1,20) = 54 p < 0.001	F(1,20) = 6 p = 0.02	F(1,20) = 96 p < 0.001	F(1,20) = 6 p = 0.02	F(1,20) = 24 p < 0.001	F(1,20) = 0 p > 0.99	F(1,20) = 0 p > 0.99
Time of groomings (s)	F(1,20) = 147 p < 0.001	F(1,20) = 75 p < 0.001	F(1,20) = 56.33 p < 0.001	F(1,20) = 56.33 p < 0.001	F(1,20) = 16.33 p < 0.001	F(1,20) = 0.33 p = 0.57	F(1,20) = 0.33 p = 0.57
Number of freezings	F(1,20) = 150 p < 0.001	F(1,20) = 150 p < 0.001	F(1,20) = 150 p < 0.001	F(1,20) = 150 p < 0.001	F(1,20) = 150 p < 0.001	F(1,20) = 150 p < 0.001	F(1,20) = 150 p < 0.001
Time of freezings (s)	F(1,20) = 150 p < 0.001	F(1,20) = 150 p < 0.001	F(1,20) = 150 p < 0.001	F(1,20) = 150 p < 0.001	F(1,20) = 150 p < 0.001	F(1,20) = 150 p < 0.001	F(1,20) = 150 p < 0.001
**Corner test**							
Number of corners	F(1,20) = 42.71 p < 0.001	F(1,20) = 0.35 p = 0.55	F(1,20) = 17.29 p < 0.001	F(1,20) = 3.18 p = 0.08	F(1,20) =3.18 p = 0.08	F(1,20) = 0.35 p = 0.55	F(1,20) = 0.35 p = 0.55
Number of wall rearings	F(1,20) = 6.86 p = 0.01	F(1,20) = 0 p > 0.99	F(1,20) = 27.43 p < 0.001	F(1,20) = 1.71 p = 0.19	F(1,20) = 1.71 p = 0.19	F(1,20) = 1.71 p = 0.19	F(1,20) = 0 p > 0.99
Number of groomings	F(1,20) = 21.43 p < 0.001	F(1,20) = 21. 43 p < 0.001	F(1,20) = 144.9 p < 0.001	F(1,20) = 7.71 p = 0.01	F(1,20) = 7.71 p = 0.01	F(1,20) = 42 p < 0.001	F(1,20) = 0.86 p = 0.36
Number of scratches	F(1,20) = 1.2 p = 0.28	F(1,20) = 10.8 p = 0.002	F(1,20) = 1.2 p = 0.28	F(1,20) = 1.2 p = 0.28	F(1,20) = 1.2 p = 0.28	F(1,20) = 1.2 p = 0.28	F(1,20) = 1.2 p = 0.28
**Burial behavior**							
Standard condition							
Number of intact pieces	F(1,20) = 145.2 p < 0.001	F(1,20) = 4.8 p = 0.03	F(1,20) = 1.2 p = 0.28	F(1,20) = 4.8 p = 0.03	F(1,20) = 1.2 p = 0.28	F(1,20) = 0 p > 0.99	F(1,20) = 0 p > 0.99
Number of moved pieces	F(1,20) = 21 p < 0.001	F(1,20) = 0.43 p = 0.52	F(1,20) = 21 p < 0.001	F(1,20) = 51.86 p < 0.001	F(1,20) = 0.43 p = 0.52	F(1,20) = 10.71 p = 0.002	F(1,20) = 0.43 p = 0.52
Number of buried pieces	F(1,20) = 66 p < 0.001	F(1,20) = 0.55 p = 0.46	F(1,20) = 4.91 p = 0.03	F(1,20) = 4.91 p = 0.03	F(1,20) = 0.55 p = 0.46	F(1,20) = 0.55 p = 0.46	F(1,20) = 0.55 p = 0.46
Bizonal condition							
Number of intact pieces	F(1,20) = 106.9 p < 0.001	F(1,20) = 19.64 p < 0.001	F(1,20) = 8.73 p = 0.005	F(1,20) = 8.73 p = 0.005	F(1,20) = 2.18 p = 0.15	F(1,20) = 2.18 p = 0.15	F(1,20) = 1 p > 0.99
Number of moved pieces	F(1,20) = 50.82 p < 0.001	F(1,20) = 5.65 p = 0.02	F(1,20) = 5.65 p = 0.02	F(1,20) = 1.41 p = 0.24	F(1,20) = 1.41 p = 0.24	F(1,20) = 1.41 p = 0.24	F(1,20) = 1 p > 0.99
Number of buried pieces	F(1,20) = 46.15 p < 0.001	F(1,20) = 7.39 p = 0.01	F(1,20) = 7.39 p = 0.01	F(1,20) = 7.39 p = 0.01	F(1,20) = 0 p > 0.99	F(1,20) = 1.85 p = 0.18	F(1,20) = 1.85 p = 0.18
**Immune function parameters**							

**Macrophage functions**							
Chemotaxis index (C.I)	$F(1,20) = 365.7$<br>$p < 0.001$	$F(1,20) = 3.86$<br>$p = 0.06$	$F(1,20) = 15.31$<br>$p < 0.001$	$F(1,20) = 4.62$<br>$p = 0.04$	$F(1,20) = 116.3$<br>$p < 0.001$	$F(1,20) = 11.55$<br>$p = 0.002$	$F(1,20) = 2.33$<br>$p = 0.14$
**Lymphocyte functions**							
Chemotaxis index (C.I)	$F(1,20) = 368$<br>$p < 0.001$	$F(1,20) = 1.57$<br>$p = 0.22$	$F(1,20) = 53.54$<br>$p < 0.001$	$F(1,20) = 0.06$<br>$p = 0.81$	$F(1,20) = 90.02$<br>$p < 0.001$	$F(1,20) = 23.86$<br>$p < 0.001$	$F(1,20) = 0.03$<br>$p = 0.87$
Natural Killer activity (%)	$F(1,20) = 14.64$<br>$p < 0.001$	$F(1,20) = 146.8$<br>$p < 0.001$	$F(1,20) = 19.93$<br>$p < 0.001$	$F(1,20) = 14.64$<br>$p < 0.001$	$F(1,20) = 104.1$<br>$p < 0.001$	$F(1,20) = 49.22$<br>$p < 0.001$	$F(1,20) = 6.51$<br>$p = 0.01$
Lymphoproliferation							
Basal proliferative response (c.p.m)	$F(1,20) = 0.96$<br>$p = 0.33$	$F(1,20) = 0.99$<br>$p = 0.33$	$F(1,20) = 43.57$<br>$p < 0.001$	$F(1,20) = 6.87$<br>$p = 0.01$	$F(1,20) = 146.3$<br>$p < 0.001$	$F(1,20) = 5.25$<br>$p = 0.03$	$F(1,20) = 0.15$<br>$p = 0.7$
Proliferative response to LPS (c.p.m)	$F(1,20) = 678.5$<br>$p < 0.001$	$F(1,20) = 1.21$<br>$p = 0.028$	$F(1,20) = 14.46$<br>$p < 0.001$	$F(1,20) = 0.24$<br>$p = 0.63$	$F(1,20) = 46.93$<br>$p < 0.001$	$F(1,20) = 3.79$<br>$p = 0.06$	$F(1,20) = 1.94$<br>$p = 0.17$
Proliferative response to ConA (c.p.m)	$F(1,20) = 426.7$<br>$p < 0.001$	$F(1,20) = 3.41$<br>$p = 0.07$	$F(1,20) = 48.65$<br>$p < 0.001$	$F(1,20) = 4.22$<br>$p = 0.05$	$F(1,20) = 82.19$<br>$p < 0.001$	$F(1,20) = 20.58$<br>$p < 0.001$	$F(1,20) = 1.16$<br>$p = 0.29$
**Oxidative stress parameters**							
**Antioxidant compounds**							
Catalase activity (UI CAT/10^6 cells)	$F(1,20) = 67.85$<br>$p < 0.001$	$F(1,20) = 1.39$<br>$p = 0.24$	$F(1,20) = 12.46$<br>$p = 0.001$	$F(1,20) = 26$<br>$p < 0.001$	$F(1,20) = 18.62$<br>$p < 0.001$	$F(1,20) = 1.39$<br>$p = 0.24$	$F(1,20) = 18.62$<br>$p < 0.001$
Glutathione reductase activity (mU GR/10^6 cells)	$F(1,20) = 28.59$<br>$p < 0.001$	$F(1,20) = 9.41$<br>$p = 0.004$	$F(1,20) = 2.22$<br>$p = 0.14$	$F(1,20) = 7.38$<br>$p = 0.01$	$F(1,20) = 12.91$<br>$p = 0.001$	$F(1,20) = 0.38$<br>$p = 0.54$	$F(1,20) = 5.6$<br>$p = 0.02$
Glutathione peroxidase activity (mU GPx/10^6 cells)	$F(1,20) = 21.49$<br>$p < 0.001$	$F(1,20) = 2.59$<br>$p = 0.11$	$F(1,20) = 89.97$<br>$p < 0.001$	$F(1,20) = 1.59$<br>$p = 0.21$	$F(1,20) = 90.71$<br>$p < 0.001$	$F(1,20) = 0.27$<br>$p = 0.6$	$F(1,20) = 0.9$<br>$p = 0.35$
Reduced glutathione levels (GSH) (nmol GSH/10^6 cells)	$F(1,20) = 1016$<br>$p < 0.001$	$F(1,20) = 50.94$<br>$p < 0.001$	$F(1,20) = 165.1$<br>$p < 0.001$	$F(1,20) = 5.66$<br>$p = 0.02$	$F(1,20) = 119.8$<br>$p < 0.001$	$F(1,20) = 0.23$<br>$p = 0.64$	$F(1,20) = 0.23$<br>$p = 0.64$
**Oxidant compounds**							
Xanthine oxidase activity (U XAO/10^6 cells)	$F(1,20) = 43.58$<br>$p < 0.001$	$F(1,20) = 2.59$<br>$p = 0.11$	$F(1,20) = 88.1$<br>$p < 0.001$	$F(1,20) = 25.66$<br>$p < 0.001$	$F(1,20) = 116.2$<br>$p < 0.001$	$F(1,20) = 29.17$<br>$p < 0.001$	$F(1,20) = 45.5$<br>$p < 0.001$
Oxidized glutathione levels (GSSG) (nmol GSSG/10^6 cells)	$F(1,20) = 129.1$<br>$p < 0.001$	$F(1,20) = 138.7$<br>$p < 0.001$	$F(1,20) = 240.1$<br>$p < 0.001$	$F(1,20) = 0.005$<br>$p = 0.95$	$F(1,20) = 138.7$<br>$p < 0.001$	$F(1,20) = 86.44$<br>$p < 0.001$	$F(1,20) = 86.44$<br>$p < 0.001$
**Redox state indicator**							
GSSG/GSH Ratio	$F(1,20) = 254.7$<br>$p < 0.001$	$F(1,20) = 61.54$<br>$p < 0.001$	$F(1,20) = 0.07$<br>$p = 0.79$	$F(1,20) = 32.27$<br>$p < 0.001$	$F(1,20) = 0.07$<br>$p = 0.79$	$F(1,20) = 0.07$<br>$p = 0.79$	$F(1,20) = 1.83$<br>$p = 0.18$

F and p values obtained from repeated measures ANOVA for each variable analyzed. The main effects of Genotype, Sex and Stress as well as the interaction between them were evaluated.
